# Meta-analysis of the therapeutic effects of different core muscle training on pain and function in patients with chronic non-specific low back pain

**DOI:** 10.3389/fpain.2026.1804665

**Published:** 2026-04-29

**Authors:** Yang Liu, Haoyang Li, Yueyong Yu, Chenyuan Zhang, Zhou Zhou

**Affiliations:** College of Physical Education and Sports, Beijing Normal University, Beijing, China

**Keywords:** breathing training, chronic non-specific low back pain, core stability training, core strength training, Pilates, sling exercise therapy

## Abstract

**Objective:**

To compare the effects of specific core muscle training modalities—core stability training, sling exercise therapy (SET), Pilates, and breathing training—vs. non-core-training controls on pain and function in patients with chronic nonspecific low back pain (CNLBP).

**Methods:**

Randomized controlled trials (RCTs) from PubMed, Web of Science, and Cochrane Library were systematically searched. Thirty-three RCTs involving 1,757 patients were analyzed using random-effects meta-analysis in RevMan, with subgroup analyses by training type, mode, and duration.

**Results:**

Core stability training significantly reduced pain (SMD = −0.95, 95% CI: −1.35 to −0.55) and improved function (SMD = −1.09, 95% CI: −1.63 to −0.55), despite high heterogeneity (*I*^2^ = 93%–95%).For pain relief, SET (SMD = −1.43) and Pilates (SMD = −1.48) showed the strongest effects, followed by breathing training (SMD = −0.75); conventional core stability training was less effective (SMD = −0.36). For functional improvement, SET (SMD = −2.60) and combined interventions (SMD = −1.07) outperformed single training. Short-term (<8 weeks) SET yielded prominent benefits, while long-term (≥8 weeks) breathing training sustained effects.

**Conclusion:**

Core muscle training alleviates pain and enhances function in CNLBP. SET and Pilates offer notable short-term analgesia, whereas combined interventions improve function more effectively. Clinical protocols should be individualized, and future studies must standardize parameters and examine multimodal synergies.

**Systematic Review Registration**: identifier: CRD420251031252.

## Introduction

Chronic nonspecific low back pain (CNLBP) ranks among the most disabling musculoskeletal disorders worldwide, posing a significant public health challenge due to its high prevalence and complex clinical course (1, 2). Epidemiological studies indicate that 50%–84% of adults experience at least one episode of low back pain in their lifetime, with 20%–25% progressing to a chronic condition (3–5). CNLBP substantially compromises quality of life and imposes a considerable socioeconomic burden through high recurrence rates and rising healthcar0e costs, making it a critical focus in rehabilitation medicine (6).

Accumulating evidence implicates dysfunction of the core musculature as a key pathomechanism in CNLBP (7, 8). The core muscles—comprising deep stabilizers such as the transversus abdominis, multifidus, diaphragm, and pelvic floor muscles—orchestrate lumbopelvic dynamic stability through coordinated contraction (9). Patients with CNLBP frequently demonstrate impaired core muscle activation timing, reduced muscular endurance, and compensatory movement patterns, leading to compromised spinal segmental stability and abnormal load distribution, thereby perpetuating a pain-dysfunction cycle (10, 11). Consequently, targeted rehabilitation of the core musculature is widely regarded as a biomechanical cornerstone for managing CNLBP (7, 8).

Within rehabilitation paradigms, core stability training (CST) has gained prominence for its role in modulating spinal function biomechanically (9). However, persistent conceptual ambiguity regarding its operational definition contributes to substantial heterogeneity in clinical research outcomes (12). Based on neuromuscular control theory, CST can be categorized into two distinct approaches: 1. Localized CST focuses on the isolated activation and endurance training of deep stabilizers (e.g., multifidus, transversus abdominis) through low-load, high-repetition static contractions to enhance segmental stability (10, 11); 2. Global functional CST integrates superficial core muscles (e.g., rectus abdominis, erector spinae) into dynamic kinetic chains, often incorporating functional movements (e.g., progressive plank variations, dynamic balance training) or external loading (e.g., suspension systems, elastic resistance) to improve whole-body postural control (7, 10). These modalities differ fundamentally in their neuroadaptive mechanisms: the former enhances local muscle recruitment timing via *γ*-motor neuron facilitation, while the latter relies on optimizing synergistic co-activation and kinetic chain efficiency (13). These two categories represent complementary approaches to core rehabilitation, which can be delivered through various specific training modalities.

Building on these neuroadaptive principles, contemporary core muscle training for CNLBP has evolved into several specific intervention modalities. The most commonly investigated include: core stability training targeting deep stabilizer activation (7, 9); sling exercise therapy (SET), which enhances neuromuscular control through unstable surfaces (14); and Pilates integrated with breathing training, combining core activation with movement pattern re-education (15). Although multiple randomized controlled trials (RCTs) confirm that core training significantly reduces pain and improves function in CNLBP patients, the comparative efficacy of different modalities remains controversial (15, 16).

Therefore, this study aims to conduct a systematic review and meta-analysis to compare the effects of different core training subtypes (core stability training, SET, Pilates, and breathing training) vs. control interventions that do not involve targeted core muscle training (e.g., usual care, general exercise, or passive therapy) on pain and functional disability in patients with CNLBP. The findings are intended to provide evidence-based guidance for clinicians in selecting optimal, personalized intervention strategies.

## Methods

### Study design

This systematic review and meta-analysis was conducted in accordance with the PRISMA guidelines (17). The study aimed to systematically evaluate and compare the therapeutic efficacy of different core muscle training modalities in improving pain and functional outcomes in patients with CNLBP.

### Research question (PICO framework)

The research question was structured according to the PICO framework as follows:

Population (P): Adults (≥18 years) diagnosed with chronic non-specific low back pain (duration ≥12 weeks).

Intervention (I): Core muscle training interventions, including core stability training, sling exercise therapy (SET), Pilates, and breathing exercises, delivered either alone or in combination with other treatments.

Comparison (C): Control interventions without targeted core muscle training (e.g., usual care, general exercise, passive therapy, or no intervention).

Outcomes (O): Pain intensity (measured by VAS or NRS) and functional disability (measured by ODI or RMDQ).

#### Inclusion and exclusion criteria

The inclusion and exclusion criteria of the literature were screened from five aspects: study type, study subjects, interventio measures, outcome indicators, and publication language, as detailed in Table 1.

**Table 1:** Inclusion and exclusion criteria for literature studies.

Project	Inclusion criteria	Exclusion criteria
Type	Randomized Controlled Trial (RCTs)	Non-randomized controlled trials(e.g., observational studies, case reports, systematic reviews, conference abstracts)
Subjects	Age ≥18 years old, meeting the diagnostic criteria of chronic nonspecific low back pain (CNLBP) (disease course ≥12 weeks)	Patients with specific low back pain, pregnancy or severe heart and lung diseases. Studies with a sample size of less than 10 cases per group (as small sample studies tend to overestimate effect sizes and lack statistical power)
Interventions	Core muscle group training (core stabilization/suspension/Pilates/breathing training), alone or in combination with other treatments	Studies that do not involve core muscle groups, or mixed interventions that cannot separate the effects of core training (e.g., concomitant surgery or drug injections)
Outcome measures	At least one of the following items should be included: 1. Pain Score (VAS/NRS) 2. Functional Score (ODI/Roland-Morris)	Outcome measures were unclear, mean ± standard deviation was not provided, or data could not be extracted

### Definitions of core muscle training modalities

To ensure consistency in intervention classification, the following operational definitions were applied:

Core Stability Training (CST): Exercises targeting the activation and strengthening of deep trunk stabilizers (e.g., transversus abdominis, multifidus) and/or superficial core muscles (e.g., rectus abdominis, erector spinae), typically performed on stable surfaces using body weight or external resistance. Examples include abdominal bracing, drawing-in maneuvers, planks, and bridging exercises.

Sling Exercise Therapy (SET): Exercises performed using suspension systems (e.g., Redcord, TerapiMaster) that create an unstable environment to challenge neuromuscular control and activate deep stabilizers. Exercises are typically performed with the body partially supported by slings, allowing for oscillatory movements and progressive resistance.

Pilates: A mind-body exercise system emphasizing core strength, postural alignment, breathing coordination, and controlled movements. Interventions could be mat-based or equipment-based (e.g., reformer, Cadillac) and were included regardless of the specific Pilates school or style.

Breathing Training: Exercises specifically targeting respiratory muscles, including diaphragmatic breathing, inspiratory muscle training (IMT), or breathing pattern re-education. These could be delivered as standalone interventions or integrated with other core training modalities.

Studies were classified into these categories based on the primary intervention described. For studies combining multiple modalities, the primary intervention was determined by the predominant training focus as reported by the authors.

### Search strategy

This study employed a standardized systematic review search methodology to comprehensively gather relevant evidence through multi-database searches. The English literature search covered three major databases: PubMed, Cochrane Library, and Web of Science, while Chinese literature was retrieved from the China National Knowledge Infrastructure (CNKI), Wanfang Data, the VIP Chinese Journal Database (VIP).

Additional searches were conducted in grey literature databases including ClinicalTrials.gov, WHO International Clinical Trials Registry Platform, and OpenGrey to minimize publication bias. Based on the PICOS framework, the search strategy combined controlled vocabulary (MeSH/Emtree terms) and free-text terms, incorporating key search terms such as “chronic nonspecific low back pain” or “CNLBP” for the condition, “core stability training,” “sling exercise therapy,” “Pilates,” and “breathing exercises” for interventions, along with “randomized controlled trial” or “RCT” as study design filters. Taking PubMed as an example, the specific search syntax was: #1 “Low Back Pain”[MeSH] OR “Chronic Non-specific Low Back Pain”[tiab] OR “CNLBP”[tiab]; #2 “Core Stability Training”[tiab] OR “Sling Exercise Therapy”[tiab] OR “Pilates”[tiab] OR “Breathing Exercise*”[tiab]; #3 “Randomized Controlled Trial”[pt] OR “RCT”[tiab]; #4 #1 AND #2 AND #3. This comprehensive search strategy was designed to ensure thorough coverage of relevant studies and maintain the integrity of the systematic review.

### Study selection process

**Figure 1:**
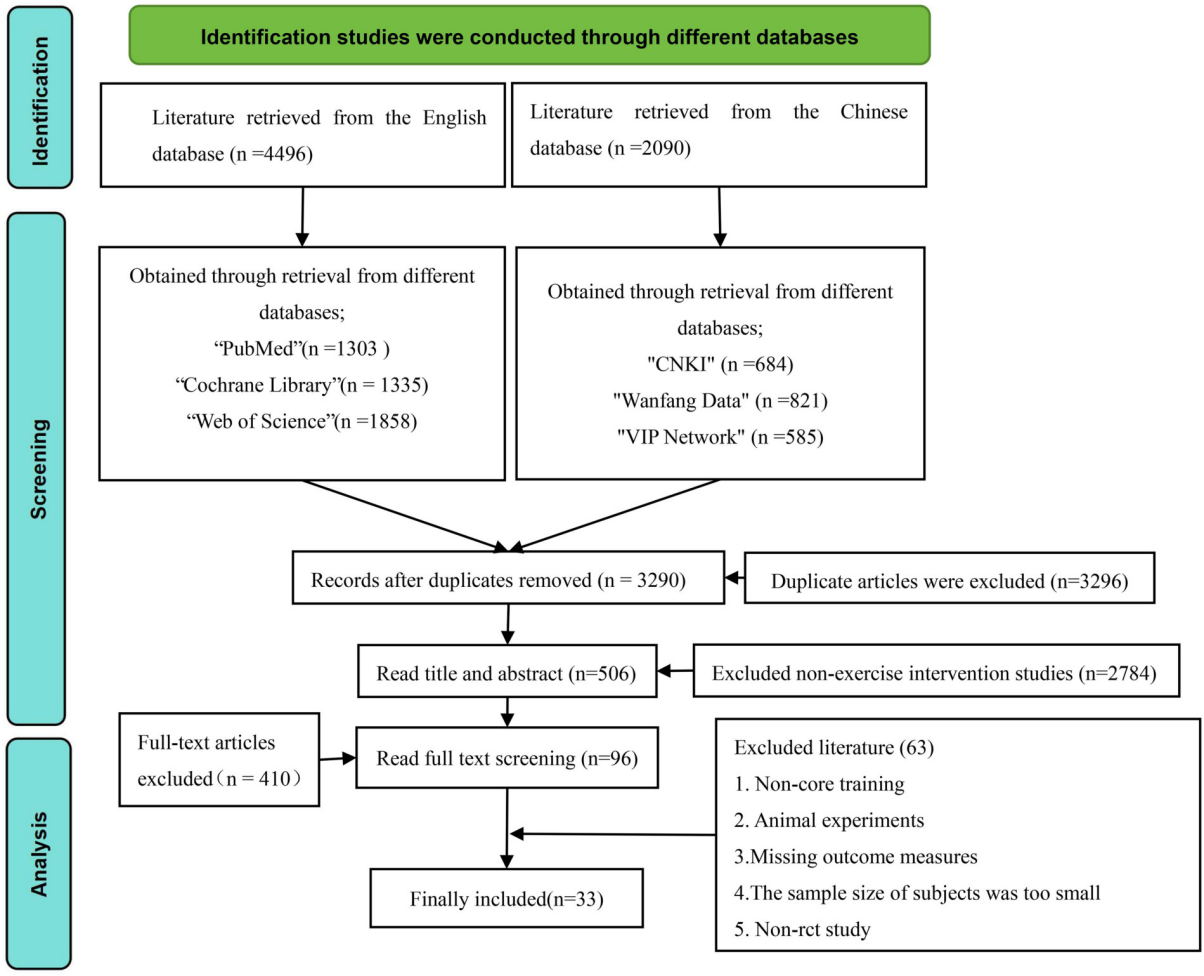
Flowchart of literature screening.

The retrieved records were imported into reference management software (e.g., EndNote X9) for deduplication. The study selection was performed independently by two reviewers (YL and HYL) based on the pre-defined eligibility criteria. Initially, titles and abstracts were screened, followed by a full-text assessment of potentially eligible studies. Any disagreements between the two reviewers at any stage were resolved through discussion; if consensus could not be reached, a third senior reviewer (YYY) was consulted to make the final decision. The selection process was documented and presented in a PRISMA flow diagram (Figure 1).

### Data extraction

A standardized data extraction form was employed in this study, and the processes of information collection and cross-validation were independently conducted by two researchers. The extracted content encompassed the author's name, publication year, country of origin, sample size, subjects' age, intervention measures for both the experimental and control groups, duration of the intervention, and outcome index data.

### Outcome assessment time points

For each included study, outcome data were extracted at baseline and at the post-intervention assessment. When multiple follow-up time points were reported, the assessment closest to the end of the intervention period was used for the primary meta-analysis. For studies reporting both short-term (<8 weeks) and long-term (≥8 weeks) follow-ups, data were extracted separately for subgroup analyses based on intervention duration.

### Data synthesis and analysis

Effect Measures and Data Handling: For continuous outcomes (pain intensity and functional disability), the mean difference (MD) and standard deviation (SD) between post-intervention values in the experimental and control groups were extracted. Since outcomes were measured using different scales (e.g., VAS, NRS for pain; ODI, RMDQ for function), the standardized mean difference (SMD) with 95% confidence interval (CI) was calculated as the summary effect measure, using Hedges' *g* to correct for small sample bias. For studies with multiple intervention groups sharing a common control, the sample size of the control group was split to avoid double-counting in pairwise comparisons.

Assessment of Heterogeneity: Statistical heterogeneity among the included studies was quantified using the *I*^2^ statistic, with values of 25%, 50%, and 75% interpreted as low, moderate, and high heterogeneity, respectively. The Cochran's *Q* test (Chi^2^) was also performed, with a significance level of *p* < 0.10 indicating significant heterogeneity.

Assessment of Reporting Biases: Potential publication bias was assessed visually through funnel plot asymmetry and statistically using Egger's linear regression test when more than 10 studies were included in a meta-analysis. The trim-and-fill method was applied to estimate the effect of potential missing studies on the pooled estimate.

Subgroup and Sensitivity Analysis: Pre-specified subgroup analyses were conducted to explore sources of heterogeneity based on: (1) type of core training (CST, SET, Pilates, breathing); (2) intervention modality (single vs. combined); (3) intervention duration (<8 vs. ≥8 weeks). Sensitivity analyses were performed by excluding studies with a high overall risk of bias to test the robustness of the results. Software: All statistical analyses were performed using Review Manager (RevMan) version 5.4 and MATLAB (R2024a).

The overall certainty (quality) of the evidence for each primary outcome (pain and function) was evaluated using the Grading of Recommendations Assessment, Development and Evaluation (GRADE) approach. Evidence from RCTs starts as high certainty but can be downgraded based on five factors: risk of bias, inconsistency, indirectness, imprecision, and publication bias. The certainty was adjudicated as high, moderate, low, or very low.

## Results

### Literature screening process and results

A total of 6,550 records were identified from database searches (2,054 from Chinese databases, 4,496 from English databases). After removing 3,260 duplicates, 3,290 records remained. Following title and abstract screening, 2,784 records were excluded, leaving 506 articles for full-text review. Full-text assessment led to the exclusion of 410 articles. Among the remaining 96 articles, 63 were excluded for reasons including non-core training interventions, missing outcome data, non-RCT designs, or insufficient sample sizes. Ultimately, 33 randomized controlled trials met the inclusion criteria and were included in the meta-analysis. The screening process is detailed in Figure 1.

### Quality assessment results of included studies

The analysis encompassed 1,757 patients with chronic nonspecific low back pain across all included studies, with 882 participants in intervention groups and 875 in control groups. The basic characteristics of the included studies are presented in Table 2.

**Table 2:** Include the basic information of the study.

Studies	The state	Sample size	Mean age (years)	Interventions		Duration	Pain outcome	Function outcome
				Experimental group	Control group			
Core stability training								
Zuo et al. (18)	China	53	28–56	CSE + IFC	CSE、IFC	12 weeks	VAS	ODI
Wang et al. (19)	China	34	18–40	PPCE	CSE	8 weeks	VAS	ODI、RMDQ
Yeldan et al. (20)	Türkiye	38	25–65	CSE + Activity training	CSE	4 weeks	VAS	RODI
Zheng et al. (21)	China	37	35.2 ± 11.1	CSE (M-Health-Based) + SCT	M-Health-Based CSE	4 weeks	NRS	RMDQ
Park et al. (22)	Korea	67	44.8 ± 10.8	SSE + ABB	SSE	24 weeks	VAS	ODI
Verbrugghe et al. (23)	Belgium	80	25–60	HITCOM、HITSTAB	Intergroup control	2weeks	NPRS	MODI
Zheng et al. (24)	China	62	35.3 ± 10.0	SCT + CSE	CSE	4 weeks	NRS	RMDQ
Duoduo Yu et al. (25)	China	60	40.43 ± 8.42	CSE + BT	CSE	8 weeks	VAS	SCODI
Park and Lee (26)	Korea	43	18–65	PPCE + BT	PPCE	4weeks	NRS	K-ODI
Oh YJ 2020 (27)	Korea	60	40–49	LSE + Breathing training	LSE	4weeks	QVAS	ODH-K
Park 2022 (28)	Korea	48	31.07 ± 6.82	LSE + Breathing training	Exercise for stability	5 weeks	QVAS	RMDQ
Sling Exercise Therapy (SET)								
Guo (29)	China	240	50.09 ± 7.67	SET	Jogging	8 weeks	VAS	ODI
Xie (30)	China	50	40–60	SET	Jogging	8 weeks	VAS	ODI
Ming 2019 (31)	China	40	38.95 ± 5.44	Electrotherapy + SET	Electrotherapy	Not explicitly mentioned	VAS	ODI
Wang et al. (32)	China	40	36.74 ± 2.68	SET	CSE	6 weeks	VAS	ODI
Li 2022 (33)	China	30	20—45	SET	Baseline control group	2weeks	-	ODI
Luo (34)	China	64	44.23 ± 10.65	SET + Pilates	SET	4 weeks	VAS	ODI
Niu et al. (35)	China	42	30.82 ± 5.2	Massage + Moxibustion + SET	Massage + Lumbar training	8 weeks	VAS	ODI
Liu et al. (36)	China	66	25∼35	SET + Rehab + Spinal correction	Rehab + Spinal correction	6 weeks	NRS	ODI
Wang et al. (37)	China	40	36.74 ± 2.68	SET	CSE	6 weeks	VAS	ODI
Pilates								
Tottoli et al. (38)	Brazil	145	18–50	Pilates	Exercise at home	6 weeks	VAS	ODI
Nageswari and Meena (39)	India	20	45–55	Pilates	Regular exercise	3 weeks	VAS	ODI
Jeon 2024 (40)	Korea	30	35.92 ± 1.6	Pilates	Cushion waist stability exercises	8 weeks	VAS	ODI
Alizad 2024 (41)	Iran	30	35–65	Pilates + Kinesio	Mulligan	6 weeks	VAS	-
Merlo et al. (42)	Brazil	38	18–64	Pilates on the mat + PBMT	Pilates on the mat	8 weeks	VAS	ODI、RMDQ
Amaral 2023 (43)	Brazil	36	18–72	Pilates + CSWD	CSWD	6 weeks	VAS	-
Akram 2024 (44)	Pakistan	42	18–40	Pilates + KT	MET + KT	8 weeks	VAS	ODI
Salahuddin 2024 (45)	Pakistan	30	30–50	Pilates + Hot compress	Hot compress	4 weeks	NPRS	-
Vera-Saura 2024 (46)	Spain	67	18–65	Pilates combines physical and mental guidance	Pilates	8 weeks	VAS	RMDQ
Swetha 2020 (47)	India	30	17–25	Pilates	Feldenkrais	6 weeks	-	ODI
Breathing training								
Gholami 2019 (48)	Iran	49	18–25	IMT	Weightlifting	8 weeks	VAS	ADI
Finta et al. (49)	Hungary	52	22.31	Diaphragm training + Complex program	Complex program	8 weeks	VAS	–
Ahmadnezhad et al. (50)	Iran	47	18–25	IMT	Weightlifting	8 weeks	VAS	–

Interventions: CSE, core stability exercise; IFC, interferential current therapy; PPCE, progressive postural control exercise; SCT, self-compassion training; SSE, spine stabilization exercise; ABB, abdominal bracing; HITCOM, high-intensity cardiorespiratory + resistance + core training; HITSTAB, high-intensity cardiorespiratory + core training; BT, breathing training; LSE, lumbar stabilization exercise; SET, sling exercise therapy; PBMT, photobiomodulation therapy; CSWD, continuous shortwave diathermy; KT, kinesio taping; MET, muscle energy technique; IMT, inspiratory muscle training.

Outcome Measures (Pain): VAS, visual analogue scale; NRS, numerical rating scale; NPRS, numerical pain rating scale; QVAS, quadruple visual analogue scale.

Outcome Measures (Function): ODI, Oswestry Disability Index; RMDQ, Roland-Morris Disability Questionnaire; RODI, revised Oswestry Disability Index; MODI, modified Oswestry Disability Index; SCODI, simplified Chinese ODI; K-ODI, Korean ODI; ODI-K, Korean ODI; ADI, athlete disability index.

Other: IG, intervention group; CG, control group; NR, not reported.

All 33 included studies reported using randomization methods. However, three studies (Guo 2018, Jeon 2024, Li 2022) failed to specify their random sequence generation methods and were consequently rated as high risk in this domain. Twenty-one studies (Ahmadnezhad 2020, Amaral 2023, Duoduo Yu 2023, Finta 2018, Gholami 2019, Jeon 2024, Jiang 2019, Li 2022, Luo 2019, Merlo 2024, Nageswari 2024, Niu 2019, Oh YJ 2020, Park 2019, Park 2022, Salahuddin 2024, Swetha 2020, Wang 2022, WangX 2019, Xie 2018, Yeldan 2023) did not provide sufficient details regarding allocation concealment, resulting in an unclear risk assessment for this criterion.

**Figure 2:**
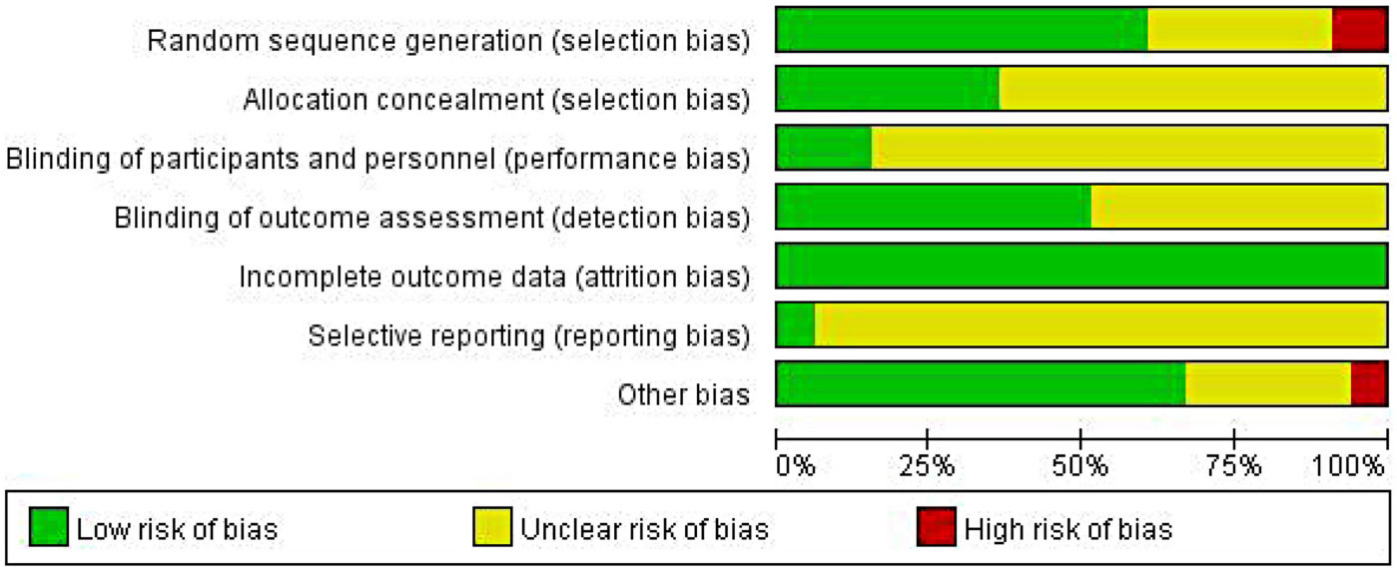
Bias risk assessment graph of the quality of the 33 included literatures.

**Figure 3:**
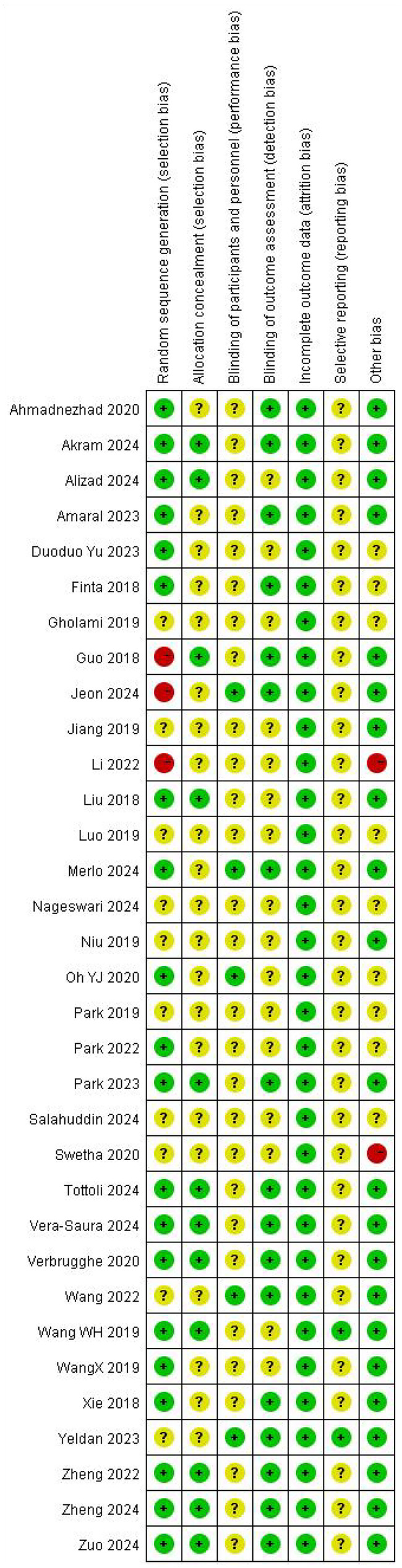
Summary plot of the risk of bias for the quality of the 33 included studies.

Five studies (Jeon 2024, Merlo 2024, Oh YJ 2020, Wang 2022, Yeldan 2023) adequately described blinding procedures and were rated as low risk for performance bias. Two studies (Wang WH 2019, Yeldan 2023) demonstrated complete outcome reporting and were assessed as low risk for selective reporting bias. All 33 studies showed low risk of attrition bias (data completeness). However, two studies (Li 2022, Swetha 2020) were ultimately classified as having high overall risk of bias. Detailed risk assessments are illustrated in Figures 2, 3.

### Risk of bias assessment

The methodological quality of the included randomized controlled trials was assessed using the Cochrane Risk of Bias Tool. Two reviewers (YL and HYL) independently evaluated seven domains: random sequence generation, allocation concealment, blinding of participants and personnel, blinding of outcome assessment, incomplete outcome data, selective reporting, and other sources of bias. Each domain was rated as low, unclear, or high risk of bias. Any disagreements were resolved through discussion or consultation with a third reviewer (YYY). The results of the risk of bias assessment are presented in Figures 2, 3.

### Results of meta-analysis

#### Pain improvement effect

**Figure 4:**
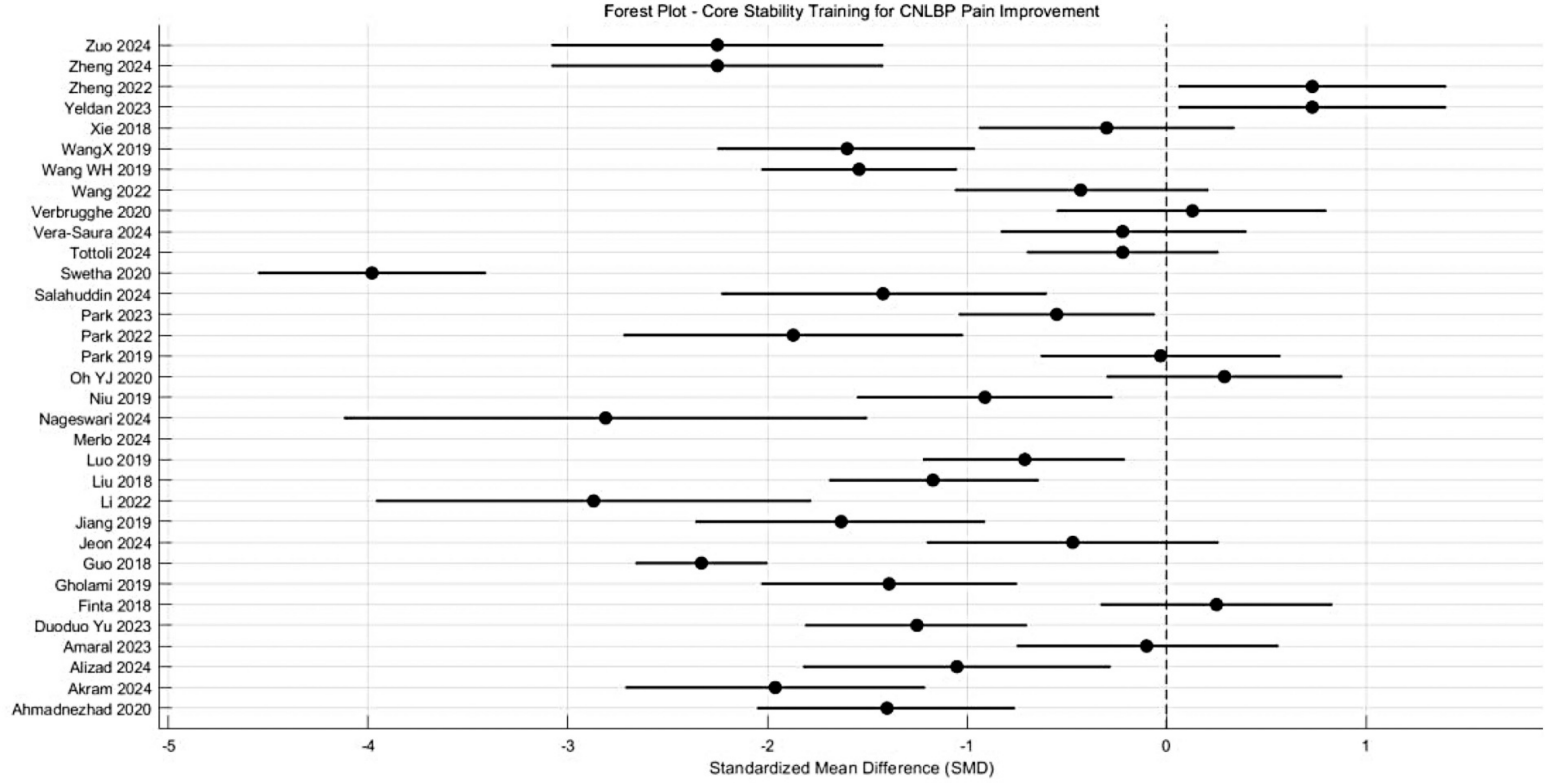
Meta-analysis forest plot of the improvement effect of core stability training on pain intensity in patients with CNLBP.

Meta-analysis of Core Stability Training for Pain Improvement in CNLBP Patients. This meta-analysis incorporated 33 randomized controlled trials involving 1,757 CNLBP patients (882 in intervention groups, 875 in controls).The pooled analysis demonstrated that core stability training yielded significantly greater pain reduction compared to control interventions (SMD = −0.95, 95% CI: −1.35 to −0.55, *P* < 0.00001), with the effect size magnitude (|SMD| = 0.95) indicating a strong treatment effect according to Cohen's d criteria (where |SMD| ≥ 0.8 represents a large effect). Notably, 85.7% of included studies (24/33) showed 95% confidence intervals not crossing the null line, further supporting the clinical superiority of the intervention (Figure 4).

Substantial heterogeneity was observed across studies (*I*^2^ = 93%), necessitating the use of a random-effects model. Two outlier studies—Swetha 2020 (SMD = −3.98) and Li 2022 (SMD = −2.87)—demonstrated markedly larger effect sizes than others, potentially attributable to their distinctive sample characteristics (e.g., Swetha 2020's unusually large control group, *n* = 73).

**Figure 5:**
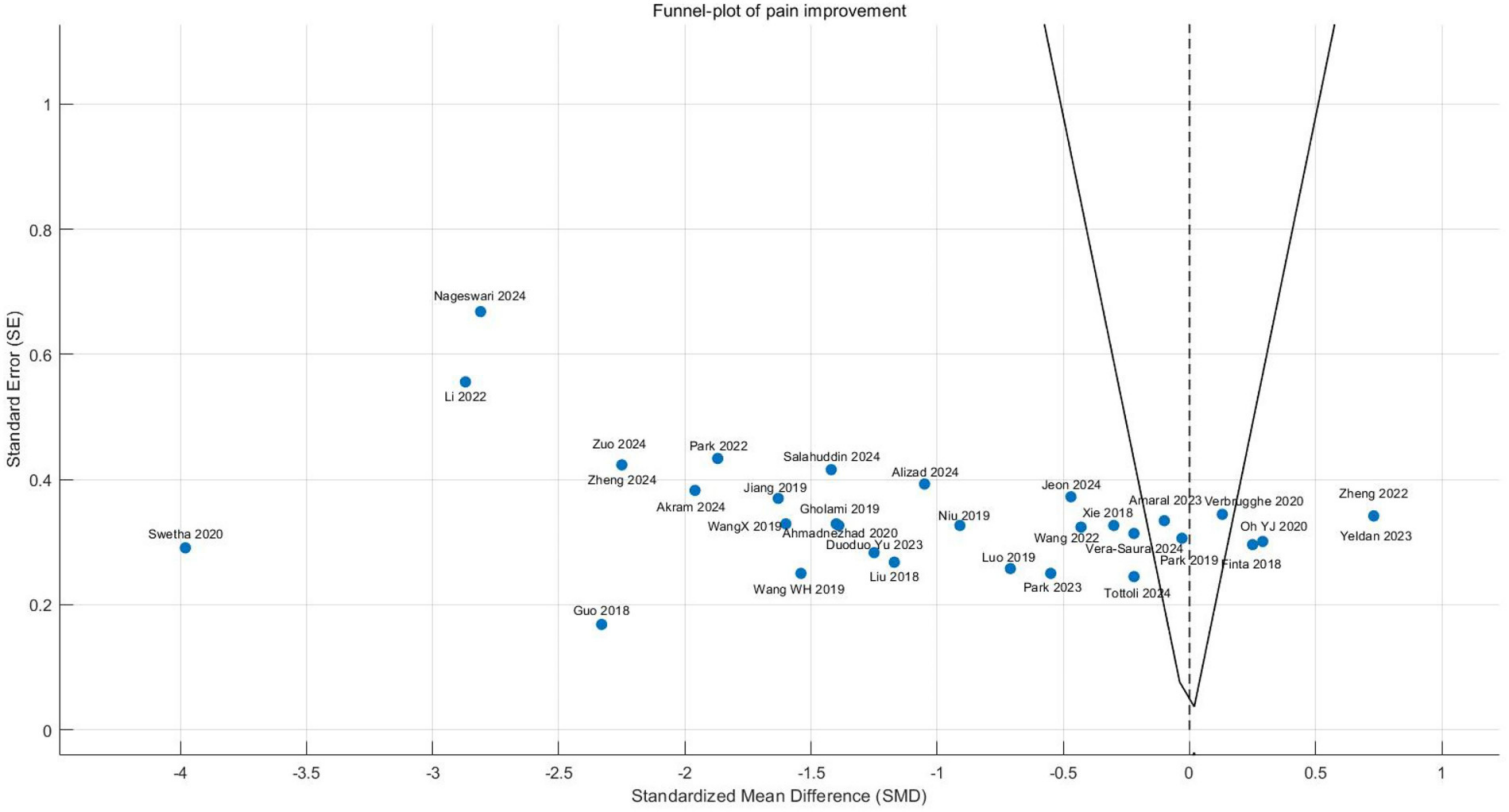
Funnel plot of meta-analysis of the effect of core stability training on pain intensity improvement in patients with CNLBP.

Publication bias assessment revealed moderate funnel plot asymmetry**,** with smaller studies (greater standard errors) clustering in the exaggerated effect size region (SMD < −1.0). Egger's test confirmed significant small-study effects (intercept = 1.65, 95% CI: 0.42–2.88, *P* = 0.02), suggesting possible selective publication of positive results or overestimation by smaller trials. Following trim-and-fill adjustment, the effect size attenuated slightly but remained statistically and clinically significant (adjusted SMD = −0.82, 95% CI: −1.21 to −0.43, *P* < 0.0001), indicating that while publication bias may have modestly inflated the original estimate, the core findings maintain robust validity (Figure 5).

### Functional improvement effect

**Figure 6:**
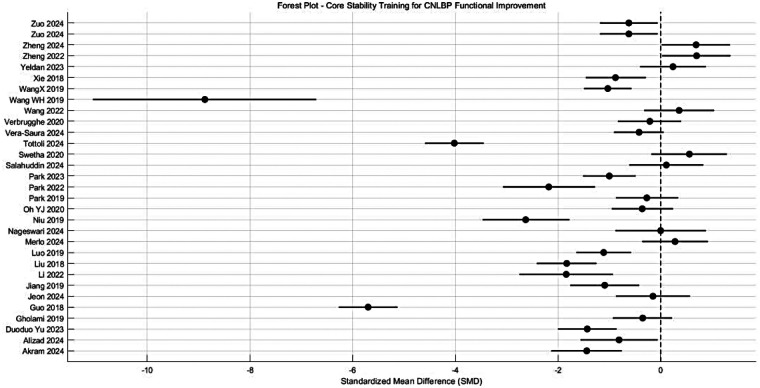
Forest plot of meta-analysis of the effects of core stability training on functional improvement in CNLBP patients.

This meta-analysis evaluated the efficacy of core stability training for functional improvement in patients with CNLBP, incorporating 30 randomized controlled trials with a total of 1,619 participants (812 in intervention groups and 807 in controls). The analysis revealed substantial between-study heterogeneity (*I*^2^ = 95%), warranting the application of a random-effects model. The pooled results demonstrated a statistically significant advantage of core stability training over control interventions in improving functional outcomes (SMD = −1.09, 95% CI: −1.63 to −0.55, *P* < 0.001). Based on Cohen's effect size criteria (where |SMD| ≥ 0.8 indicates a large effect), the magnitude of improvement (|SMD| = 1.09) suggests a very strong treatment effect (Figure 6).

**Figure 7:**
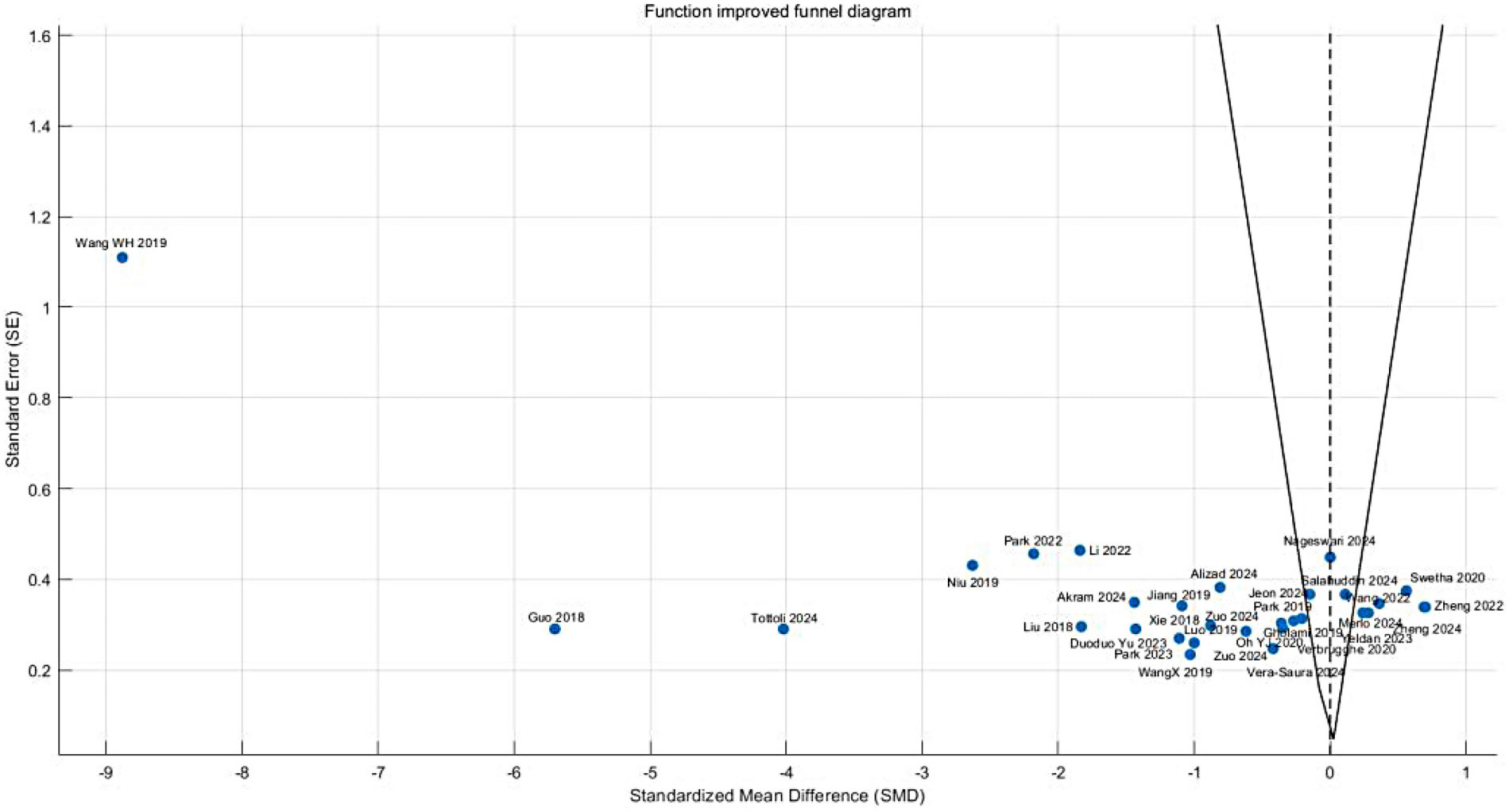
Funnel plot of a meta-analysis of the effects of core stability training on functional improvement in patients with CNLBP.

Visual inspection of the funnel plot revealed notable asymmetry, characterized by smaller studies (with larger standard errors) clustering in the region of effect size overestimation (SMD < −1.5), while larger studies were distributed around the mean effect size (SMD = −1.09). This observation was corroborated by Egger's test (intercept = 2.34, 95% CI: 1.12 to 3.56, *P* = 0.003), indicating potential publication bias that may stem from either inflated effect sizes in smaller studies or the underreporting of negative findings. Following trim-and-fill adjustment, the effect size estimate was attenuated to −0.87 (95% CI: −1.42 to −0.32, *P* = 0.002) while maintaining statistical significance. This suggests that while the initial strong effect size may have been partially influenced by bias, the adjusted results continue to support the functional benefits of core stability training for CNLBP patients (Figure 7).

## Subgroup analysis results

### Effects of different core training types (pilates vs. SET vs.) breathing training vs. core stability training

Across the included studies, control interventions varied considerably: 15 studies used conventional core exercise as control, 8 used general exercise (e.g., jogging, weightlifting), 6 used passive therapy (e.g., electrotherapy, hot compress), and 4 used usual care or waiting list controls. This variation in comparator may partly explain the differential effect sizes observed across intervention types.

#### Improvement of pain

**Figure 8:**
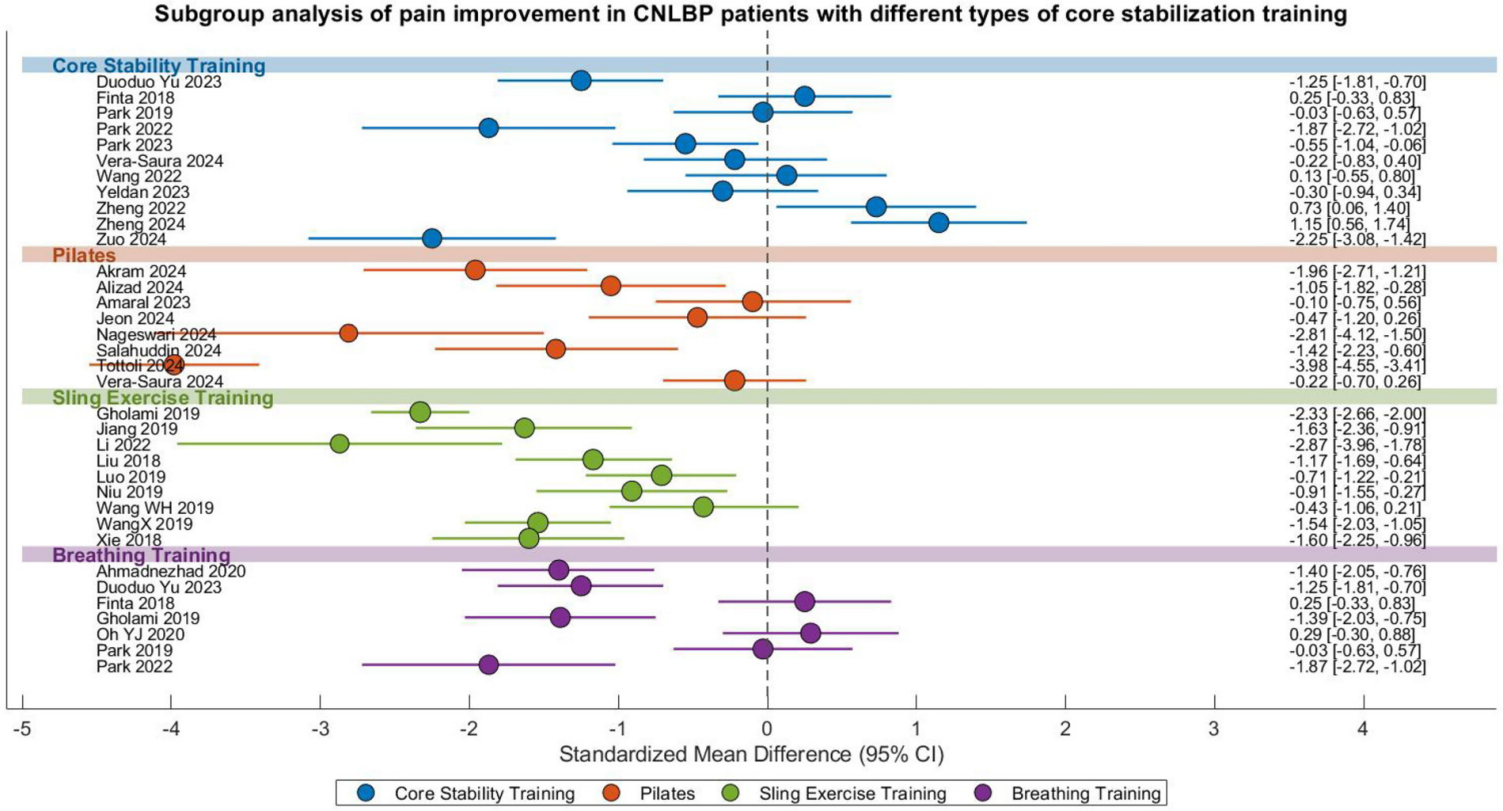
Subgroup meta-analysis forest plot of the effect of different types of core stability training on pain improvement in patients with CNLBP.

This meta-analysis evaluated the comparative effectiveness of different core stability training modalities for pain relief in CNLBP patients, incorporating 36 randomized controlled trials with a total of 1,902 participants (955 in intervention groups and 947 in control groups). The analysis revealed substantial heterogeneity across studies (*I*^2^ = 92%), necessitating the use of a random-effects model. The overall pooled effect size demonstrated statistically superior pain reduction with core stability training compared to control interventions (SMD = −0.97, 95% CI: −1.34 to −0.60, *Z* = 5.16, *P* < 0.00001), representing a strong treatment effect according to Cohen's criteria (|SMD| = 0.97) (Figure 8).

Subgroup analyses revealed differential effectiveness among intervention types (Figure 8). While conventional core stability training (11 studies) showed non-significant pain reduction (SMD = −0.36, 95% CI: −0.91 to 0.19, *P* = 0.20) with moderate-high heterogeneity (*I*^2^ = 88%), other modalities demonstrated significant benefits: Pilates (9 studies, SMD = −1.48, 95% CI: −2.55 to −0.41, *P* < 0.007, *I*^2^ = 95%), SET (9 studies, SMD = −1.43, 95% CI: −1.93 to −0.94, *P* < 0.00001, *I*^2^ = 86%), and breathing exercises (7 studies, SMD = −0.75, 95% CI: −1.41 to −0.1, *P* = 0.020, *I*^2^ = 87%). The significant between-subgroup heterogeneity (*I*^2^ = 87.9%, *P* = 0.03) likely reflects variations in intervention protocols (e.g., intensity, frequency) and outcome assessment methods.

**Figure 9:**
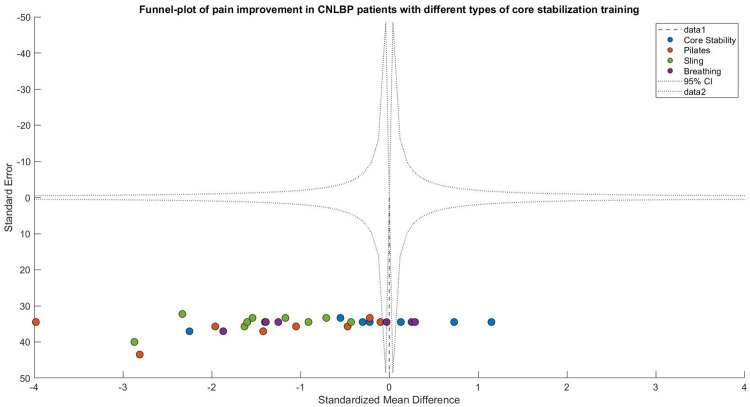
Funnel plot of the subgroup meta-analysis of the pain improvement effect of different types of core stability training on patients with CNLBP.

Funnel plot asymmetry suggested potential publication bias, with smaller studies (greater standard errors) showing exaggerated effect sizes (SMD < −1.0) compared to larger studies clustered around the mean effect (SMD = −0.97). This pattern indicates possible small-study effects or selective reporting of favorable outcomes (Figure 9).

### Functional improvement

**Figure 10:**
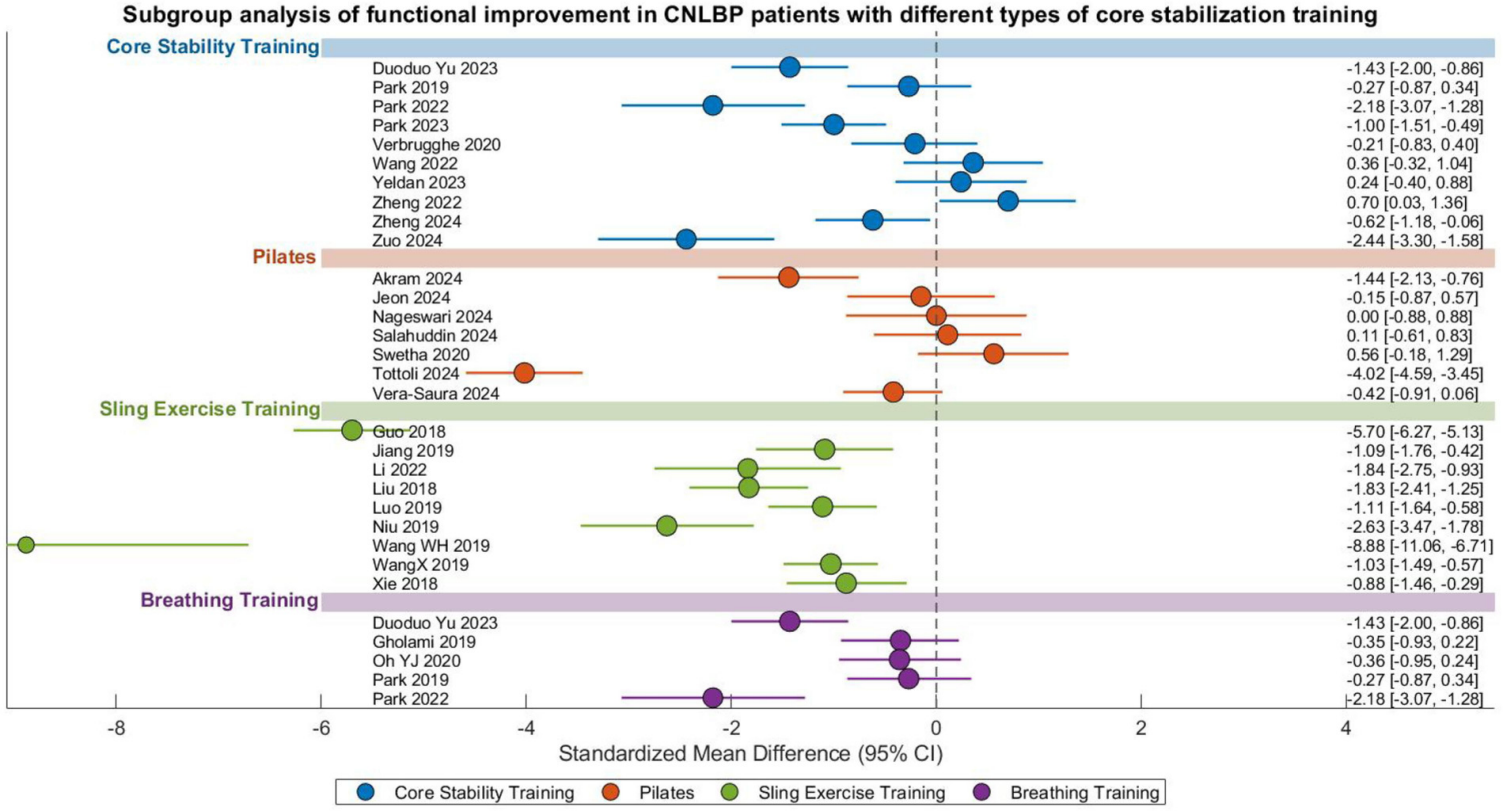
Subgroup meta-analysis forest plot of the functional improvement effect of different types of core stability training on patients with CNLBP.

This meta-analysis systematically evaluated the effects of various core stability training modalities on functional improvement in patients with CNLBP, comprising 31 randomized controlled trials with a total of 1,687 participants (844 in intervention groups and 843 in control groups). The analysis demonstrated extremely high between-study heterogeneity (*I*^2^ = 95%), justifying the application of a random-effects model. The overall pooled effect size revealed that core stability training significantly enhanced functional outcomes compared to control interventions (SMD = −1.26, 95% CI: −1.77 to −0.75, *Z* = 4.81, *P* < 0.00001), representing a strong treatment effect according to Cohen's criteria (|SMD| = 1.26) (Figure 10).

Subgroup analyses showed differential effectiveness across intervention types: conventional core stability training (10 studies) yielded moderate but significant functional improvement (SMD = −0.66, 95% CI: −1.23 to −0.09, *P* = 0.02) with considerable heterogeneity (*I*^2^ = 87%); Pilates (7 studies) demonstrated non-significant effects (SMD = −0.78, 95% CI: −2.03 to 0.48, *P* = 0.22) with extreme heterogeneity (*I*^2^ = 96%); SET (9 studies) showed the strongest effects (SMD = −2.60, 95% CI: −3.82 to −1.38, *P* < 0.0001) despite extreme heterogeneity (*I*^2^ = 97%); while breathing exercises (5 studies) produced significant benefits (SMD = −0.87, 95% CI: −1.52 to −0.22, *P* = 0.009) with high heterogeneity (*I*^2^ = 81%). The substantial between-subgroup heterogeneity (*I*^2^ = 63.3%, *P* = 0.04) likely reflects variations in intervention protocols and outcome measurement approaches.

**Figure 11:**
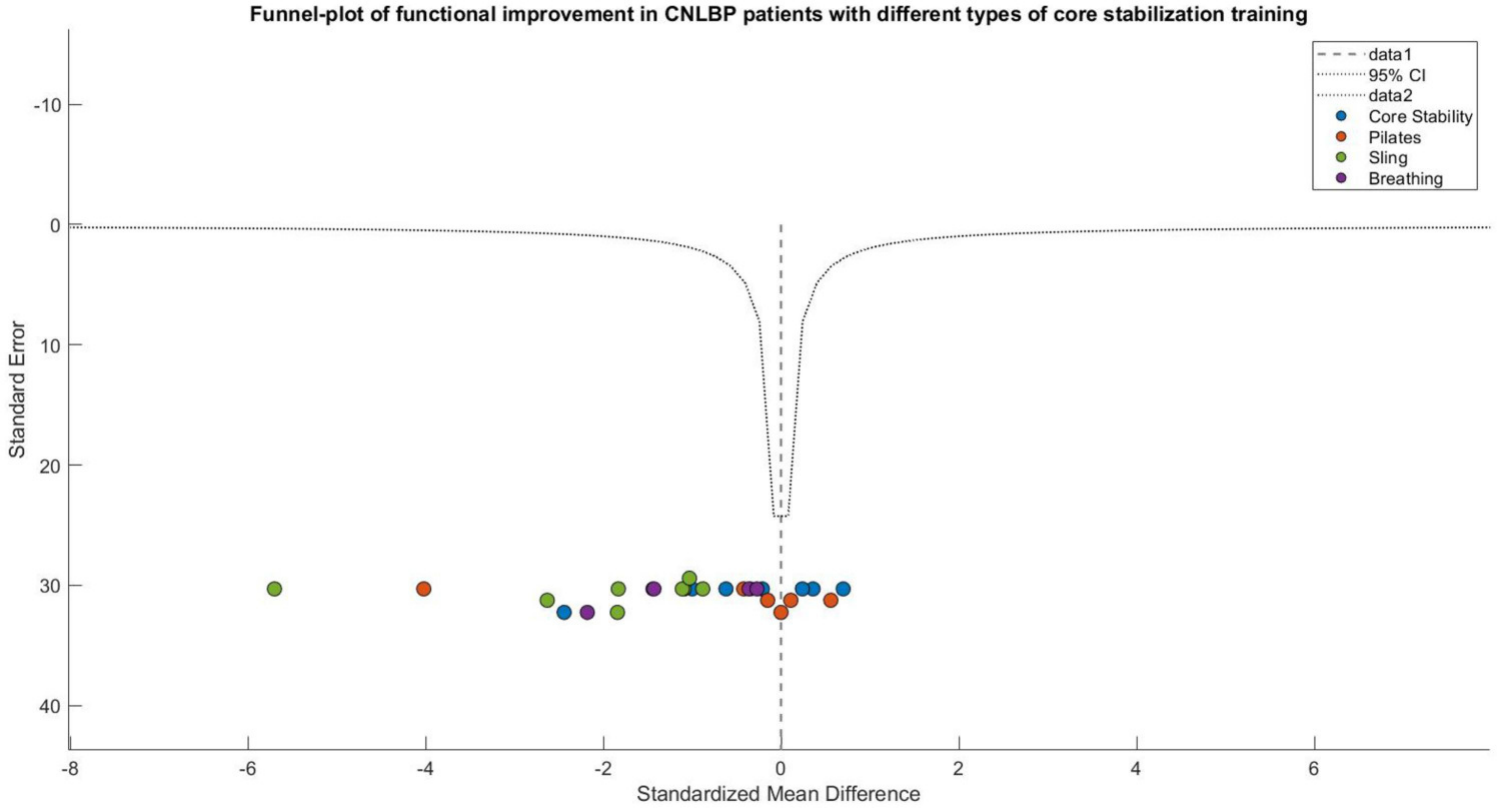
Funnel plot of the subgroup meta-analysis of the functional improvement effect of different types of core stability training on patients with CNLBP.

Funnel plot asymmetry indicated potential publication bias, with smaller studies (greater standard errors) displaying widely dispersed effect sizes (e.g., SMD = −2.60 for SET) that deviated from the overall effect (SMD = −1.26), while larger studies clustered in the high-effect region. This pattern suggests possible small-study effects or selective outcome reporting, warranting cautious interpretation of the findings (Figure 11).

#### The effects of the simple group and the combined group under the same core training type

**Simple and Combined**—**Pain**
**Core Stability Training**—**Pain**

**Figure 12:**
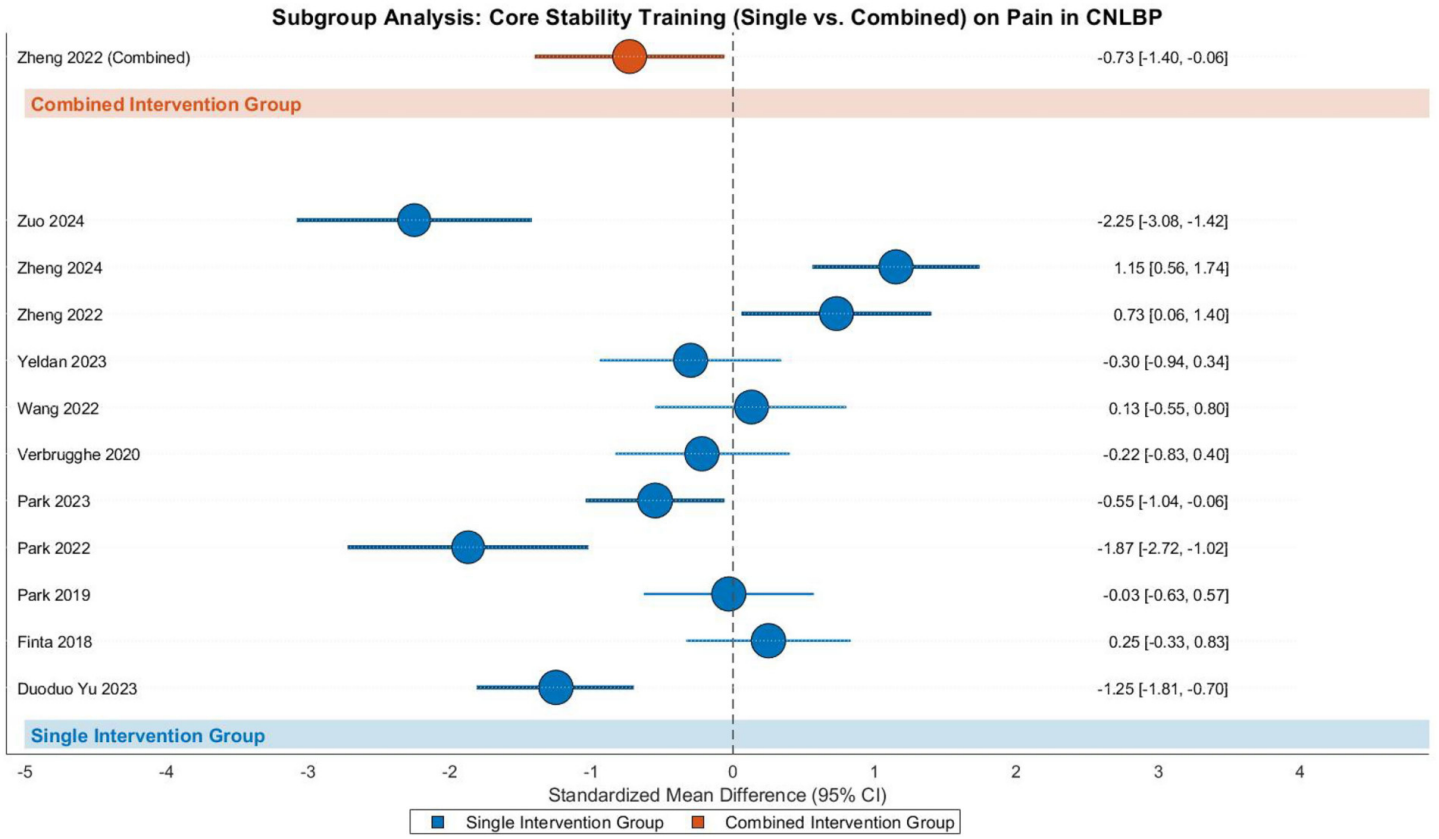
Subgroup meta-analysis forest plot of the effect of core stability training on pain improvement in patients with CNLBP.

This meta-analysis evaluated the pain-relieving effects of core stability training in patients with CNLBP through subgroup analysis of 12 randomized controlled trials (11 single-modality studies and 1 combined-intervention study), involving a total of 526 participants (262 in intervention groups and 264 in controls). The analysis revealed substantial heterogeneity across studies (Tau^2^ = 0.70, *I*^2^ = 87%, *P* < 0.00001), necessitating the use of a random-effects model. The overall pooled effect size indicated no statistically significant pain reduction with core stability training (SMD = −0.39, 95% CI: −0.90 to 0.12, *Z* = 1.49, *P* = 0.14) (Figure 12).

Subgroup analysis demonstrated that single-modality core stability training (11 studies) showed non-significant pain improvement (SMD = −0.36, 95% CI: −0.91 to 0.19, *Z* = 1.28, *P* = 0.20) with considerable heterogeneity (Tau^2^ = 0.76, *I*^2^ = 88%, *P* < 0.00001). In contrast, the combined intervention group (1 study) exhibited significant pain reduction (SMD = −0.73, 95% CI: −1.40 to −0.06, *Z* = 2.15, *P* = 0.03), though this finding should be interpreted cautiously due to the single-study nature of this subgroup. Notably, between-subgroup comparisons revealed no statistically significant difference in treatment effects (Chi^2^ = 0.71, df = 1, *P* = 0.40, *I*^2^ = 0%), suggesting comparable efficacy between single and combined intervention approaches.

**Figure 13:**
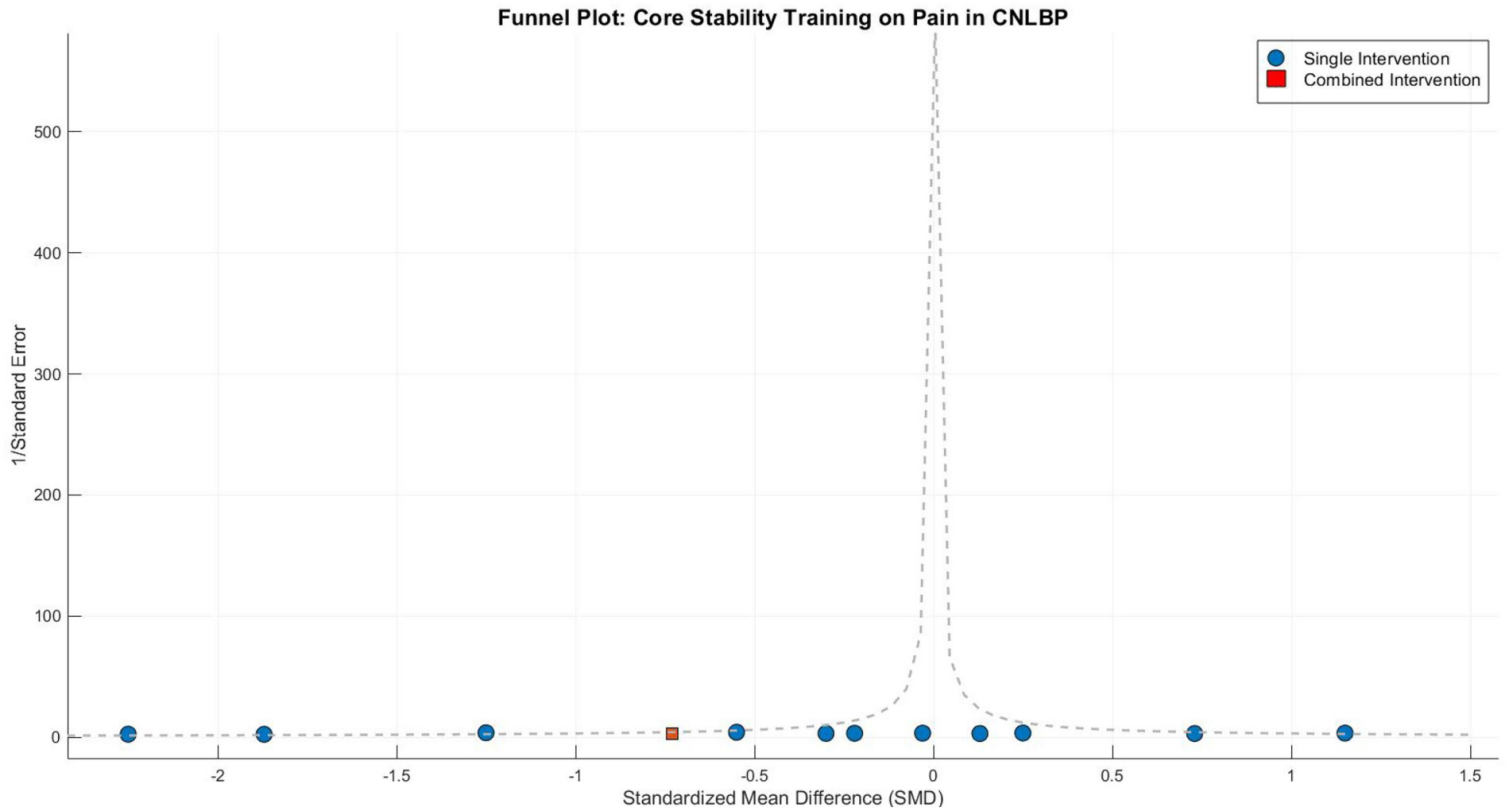
Funnel plot of the subgroup meta-analysis of the pain improvement effect of core stability training on patients with CNLBP.

The funnel plot displayed marked asymmetry, with smaller studies (exhibiting greater standard errors) clustered in the region of larger effect sizes (e.g., SMD = −0.73 for the combined intervention), while larger studies were distributed closer to the line of no effect (as evidenced by the 95% CI of the single-modality group encompassing zero). This pattern raises concerns about potential publication bias or small-study effects that may have influenced the overall findings (Figure 13).

**SET- Pain**

**Figure 14:**
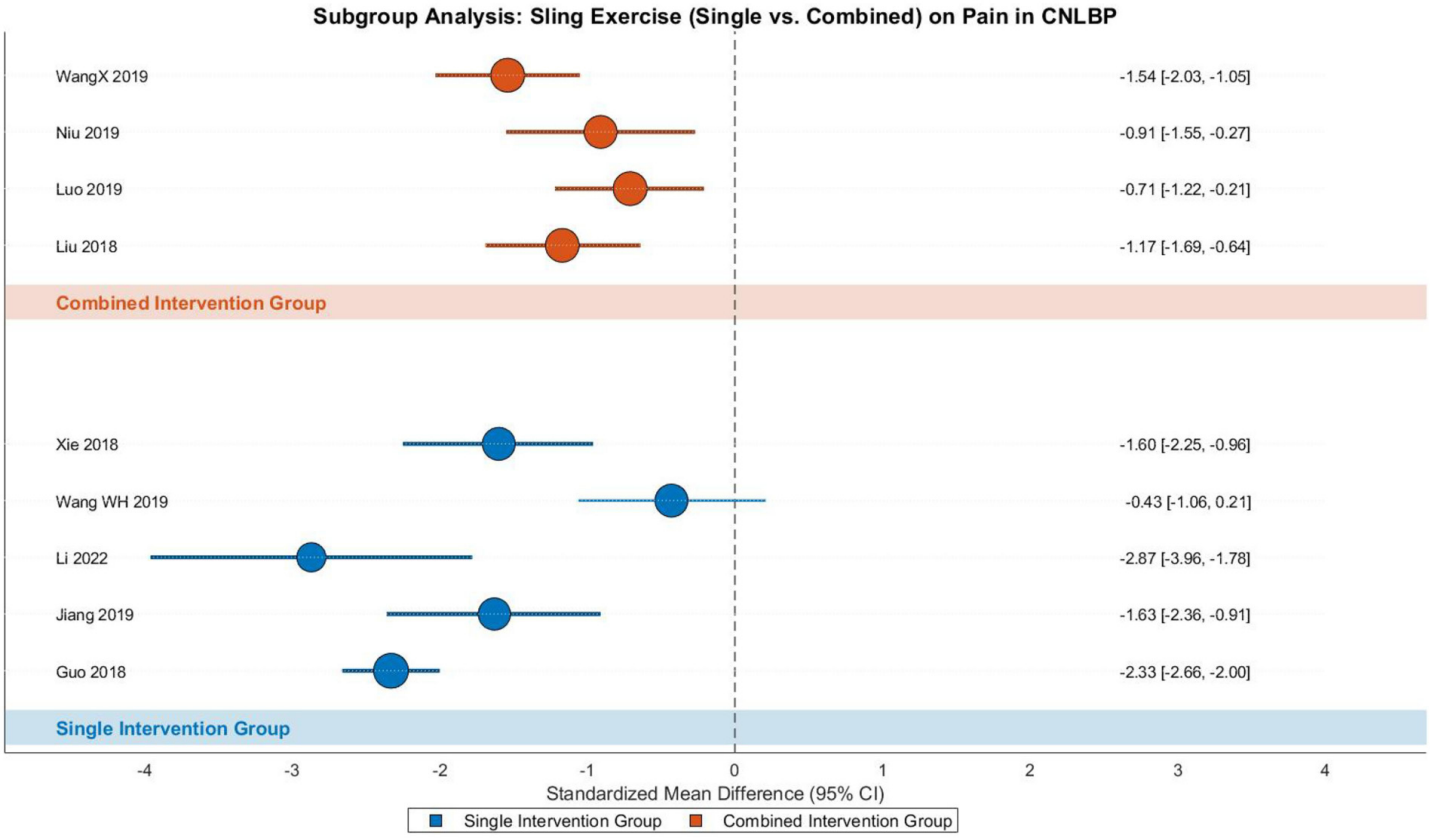
Subgroup meta-analysis forest plot of the effect of SET on pain improvement in patients with CNLBP.

This meta-analysis examined the pain-relieving effects of SET in CNLBP patients through subgroup analysis of 9 randomized controlled trials (5 single-modality studies and 4 combined-intervention studies), involving 655 participants (332 in intervention groups and 323 in controls). The analysis demonstrated substantial heterogeneity across studies (Tau^2^ = 0.48, *I*^2^ = 86%, *P* < 0.00001), warranting the use of a random-effects model. The pooled results revealed that SET produced significantly greater pain reduction compared to control interventions (SMD = −1.43, 95% CI: −1.93 to −0.94, *Z* = 5.65, *P* < 0.00001), representing a strong treatment effect according to Cohen's criteria (|SMD| ≥ 0.8) (Figure 14).

Subgroup analysis showed that both single-modality SET (5 studies) and combined interventions (4 studies) yielded significant pain improvement. The single-modality subgroup demonstrated a strong effect size (SMD = −1.74, 95% CI: −2.52 to −0.96, *Z* = 4.38, *P* < 0.0001) with considerable heterogeneity (Tau^2^ = 0.66, *I*^2^ = 87%, *P* < 0.00001), while the combined intervention subgroup also showed a strong effect (SMD = −1.10, 95% CI: −1.46 to −0.73, *Z* = 5.81, *P* < 0.00001) with moderate heterogeneity (Tau^2^ = 0.07, *I*^2^ = 47%, *P* = 0.13). Between-subgroup comparisons indicated no statistically significant difference in treatment effects (Chi^2^ = 2.15, df = 1, *P* = 0.14, *I*^2^ = 53.5%), suggesting comparable efficacy between the two approaches.

**Figure 15:**
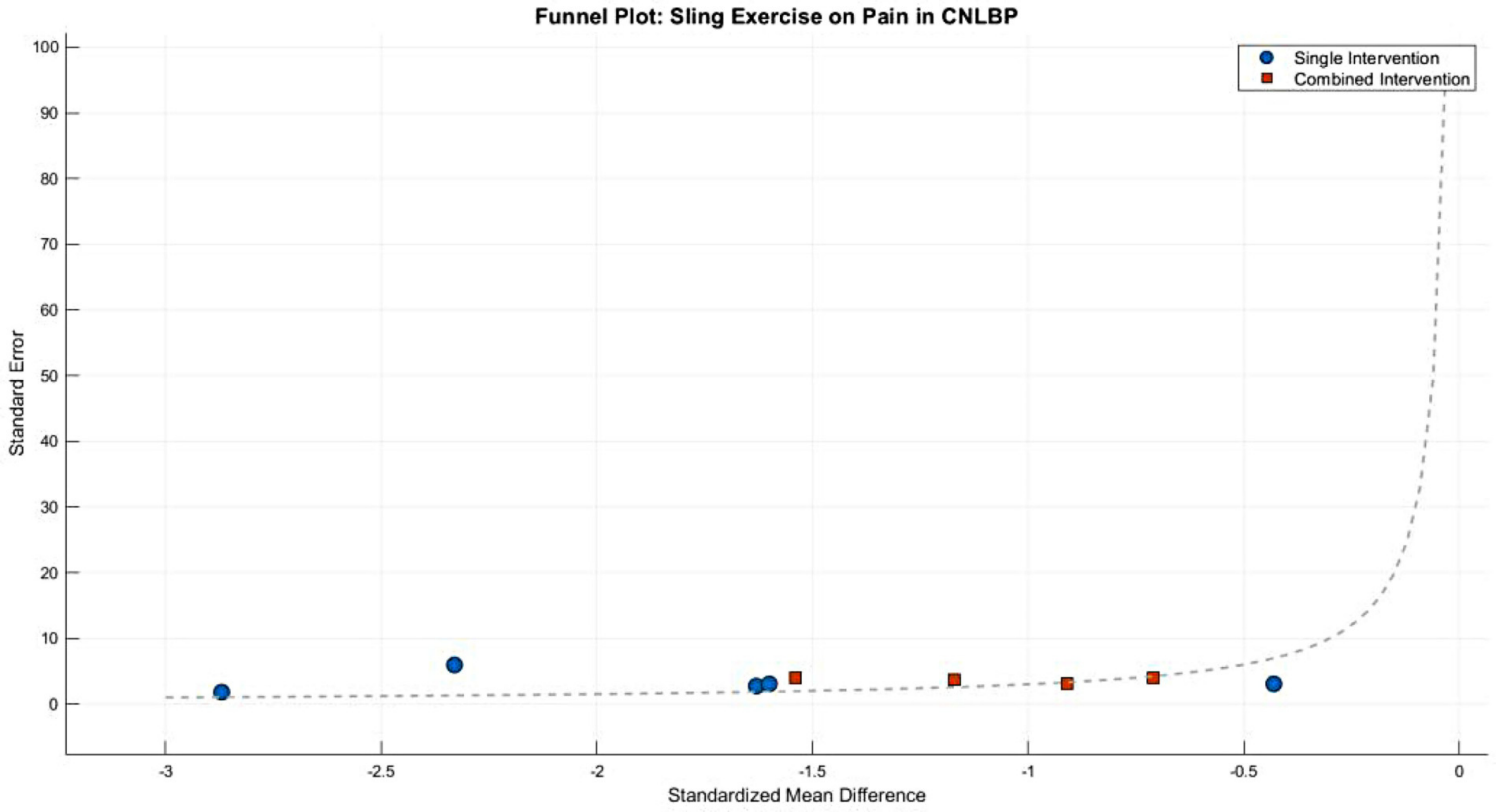
Funnel plot of the subgroup meta-analysis of the pain improvement effect of SET on patients with CNLBP.

Funnel plot analysis revealed moderate asymmetry, with smaller studies (showing greater standard errors) distributed toward more extreme effect sizes (e.g., SMD = −1.74 in the single-modality group), while larger studies clustered around the mean effect size (SMD = −1.43). Notably, some studies in the single-modality subgroup demonstrated particularly extreme effect estimates (approaching SMD = −2.52), potentially reflecting methodological variations or other study-specific factors influencing the outcomes (Figure 15).

**Pilates**—**Pain**

**Figure 16:**
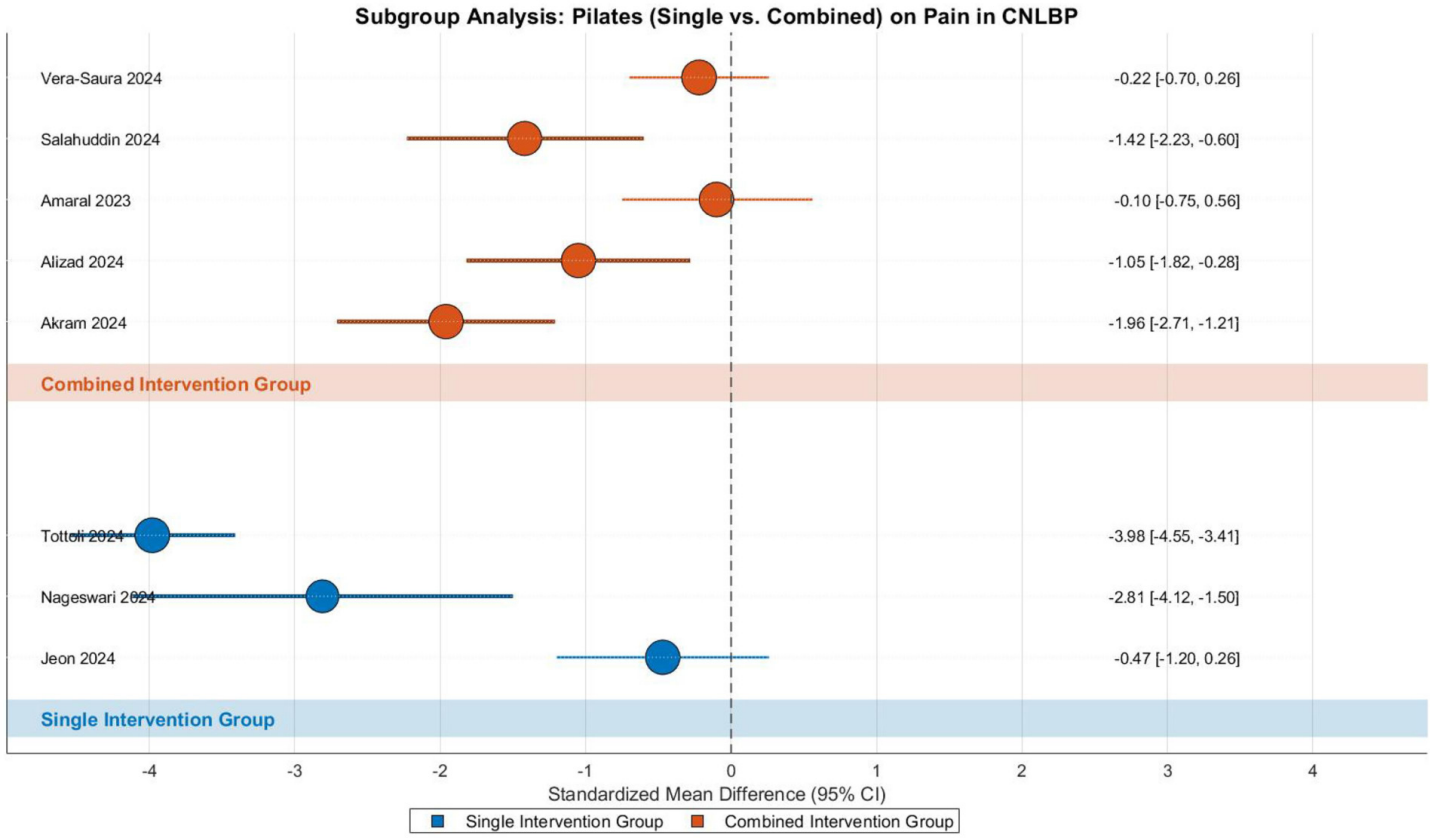
Subgroup meta-analysis forest plot of the effect of Pilates on pain improvement in patients with CNLBP.

This meta-analysis evaluated the efficacy of Pilates training for pain relief in CNLBP patients through subgroup analysis of 9 randomized controlled trials (3 single-modality studies and 6 combined-intervention studies), involving 438 participants (219 in each group). The analysis revealed extreme heterogeneity across studies (Tau^2^ = 2.23, *I*^2^ = 95%, *P* < 0.00001), necessitating the use of a random-effects model. The pooled results demonstrated that Pilates training produced significantly greater pain reduction compared to control interventions (SMD = −1.48, 95% CI: −2.55 to −0.41, *Z* = 2.71, *P* = 0.007), representing a strong treatment effect according to Cohen's criteria (Figure 16).

Subgroup analysis showed differential patterns between intervention types. The single-modality subgroup (3 studies) approached statistical significance (SMD = −2.42, 95% CI: −4.86 to 0.02, *Z* = 1.94, *P* = 0.05) with extreme heterogeneity (Tau^2^ = 4.43, *I*^2^ = 96%), where the Tottoli 2024 study (SMD = −3.98) substantially influenced the effect size, potentially due to its large sample size (*N* = 145) and rigorous training protocol. The combined-intervention subgroup (5 studies) demonstrated moderate-strong effects (SMD = −0.92, 95% CI: −1.62 to −0.21, *Z* = 2.54, *P* = 0.01) with high heterogeneity (Tau^2^ = 0.52, *I*^2^ = 82%). Notably, the Merlo2024 study was excluded from pooled analysis due to zero standard deviation in the control group. Between-subgroup comparisons revealed no statistically significant difference (Chi^2^ = 1.34, df = 1, *P* = 0.25, *I*^2^ = 25.5%), suggesting comparable pain-relieving effects between single and combined approaches.

**Figure 17:**
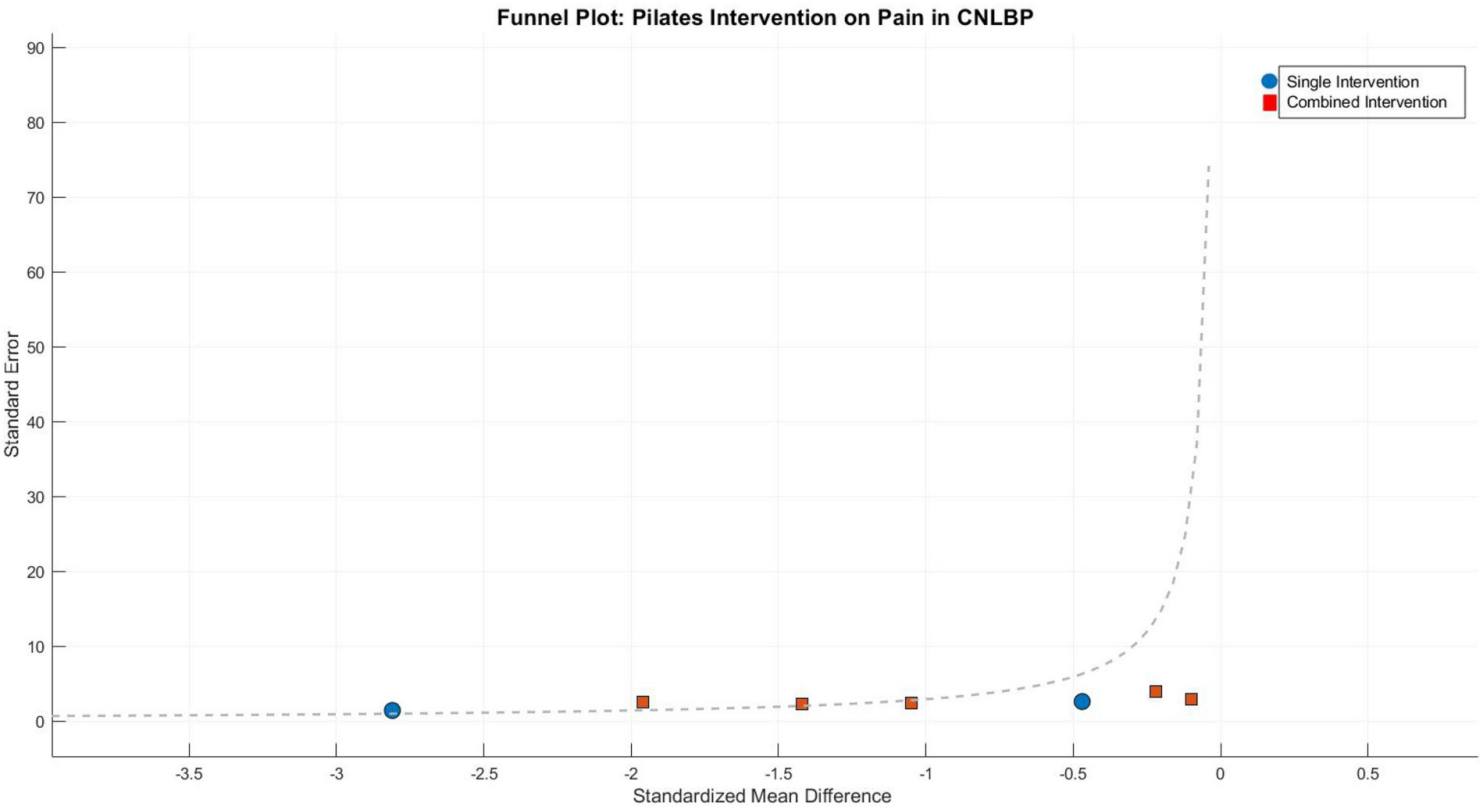
Funnel plot of the subgroup meta-analysis of the pain improvement effect of Pilates on patients with CNLBP.

Funnel plot asymmetry indicated potential bias, with smaller studies showing widely dispersed effect sizes and the single-modality subgroup containing extreme values (e.g., Tottoli 2024, SMD = −3.98) that substantially inflated the overall effect estimate, while larger studies clustered around the mean effect size (SMD = −1.48). This pattern warrants cautious interpretation of the findings (Figure 17).

**Breathing Training**—**Pain**

**Figure 18:**
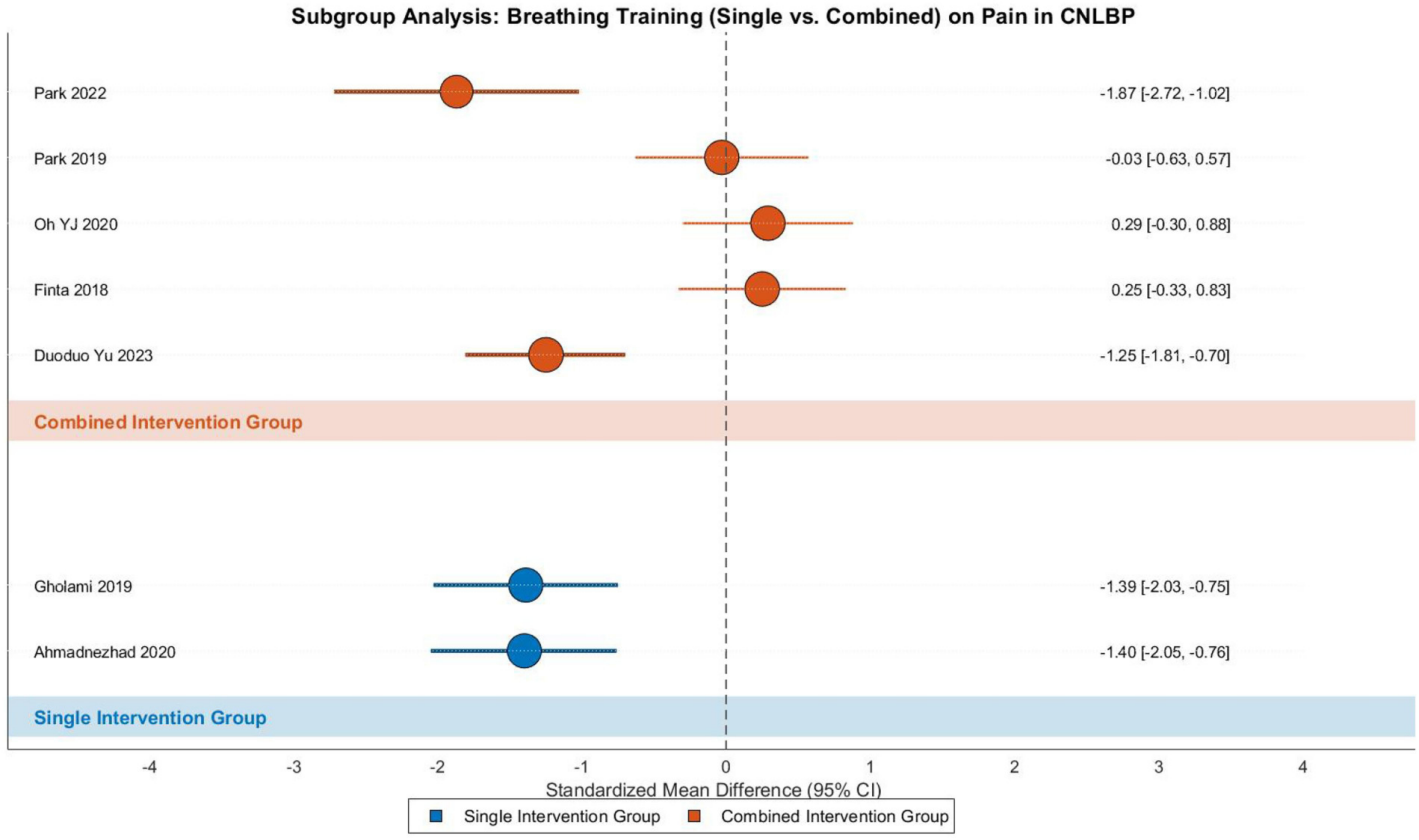
Subgroup meta-analysis forest plot of the effect of respiratory training on pain improvement in patients with CNLBP.

This meta-analysis examined the pain-relieving effects of breathing exercises in CNLBP through subgroup analysis of 7 randomized controlled trials (2 single-modality studies and 5 combined-intervention studies), involving 320 participants (161 in intervention groups and 159 in controls). The analysis demonstrated substantial heterogeneity across studies (Tau^2^ = 0.67, *I*^2^ = 87%, *P* < 0.00001), justifying the use of a random-effects model. The pooled results indicated that breathing exercises produced statistically significant pain reduction (SMD = −0.75, 95% CI: −1.41 to −0.10, *Z* = 2.25, *P* = 0.02), representing a moderate treatment effect according to Cohen's criteria (Figure 18).

Subgroup analysis revealed distinct patterns: the single-modality subgroup (2 studies) showed consistent strong effects (SMD = −1.40, 95% CI: −1.85 to −0.94, *Z* = 6.02, *P* < 0.00001) with minimal heterogeneity (Tau^2^ = 0.00, *I*^2^ = 0%), where both studies (Ahmadnezhad 2020 and Gholami 2019) demonstrated remarkably similar effect sizes (SMD ≈ −1.40), suggesting robust pain-relieving effects of standalone breathing exercises. In contrast, the combined-intervention subgroup (5 studies) showed non-significant results (SMD = −0.50, 95% CI: −1.28 to 0.29, *Z* = 1.23, *P* = 0.22) with considerable heterogeneity (Tau^2^ = 0.70, *I*^2^ = 88%). The divergent outcomes in this subgroup—ranging from Park 2022 (SMD = −1.87) showing strong effects to Finta 2018 (SMD = 0.25) and Oh YJ 2020 (SMD = 0.29) demonstrating opposite trends—likely reflect the variability in combined intervention protocols (e.g., integration with core training or exercise therapy).

**Figure 19:**
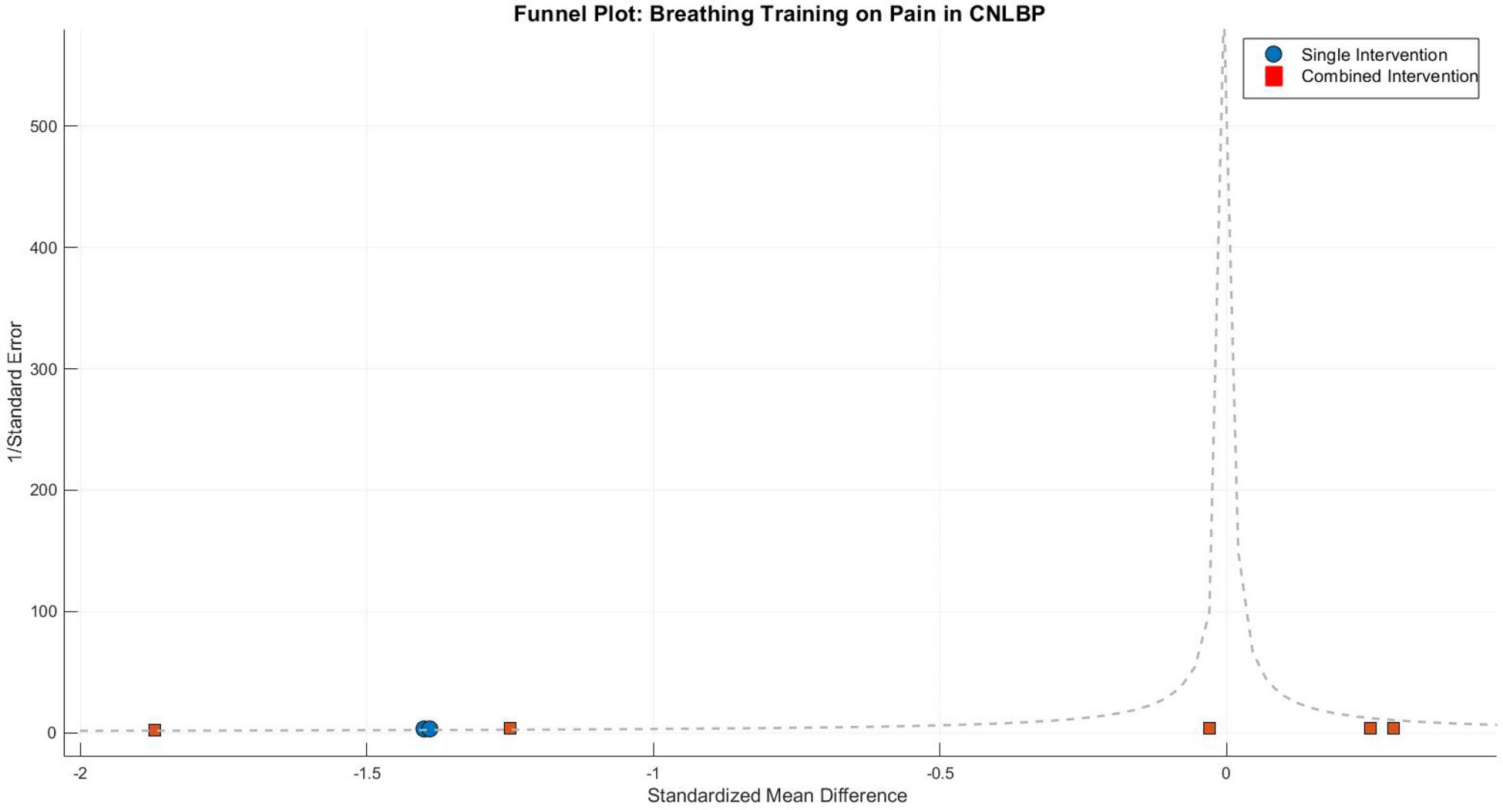
Funnel plot of the subgroup meta-analysis of the pain improvement effect of respiratory training on patients with CNLBP.

Between-subgroup comparisons approached statistical significance (Chi^2^ = 3.77, df = 1, *P* = 0.05, *I*^2^ = 73.5%), suggesting potential fundamental differences in pain-relieving mechanisms between standalone and combined breathing exercise approaches. Funnel plot asymmetry revealed distinct patterns: the two single-modality studies clustered tightly in the strong-effect region (SMD ≈ −1.40), while the combined-intervention studies showed wide dispersion, with both extreme positive effects (Park 2022, SMD = −1.87) and counterintuitive negative effects (Finta 2018, SMD = 0.25). This distribution underscores how the high heterogeneity (*I*^2^ = 88%) in combined interventions may stem from substantial variations in treatment protocols and adjunct therapies (Figure 19).

### Simplicity and combination—function

#### Core stability training—function

**Figure 20:**
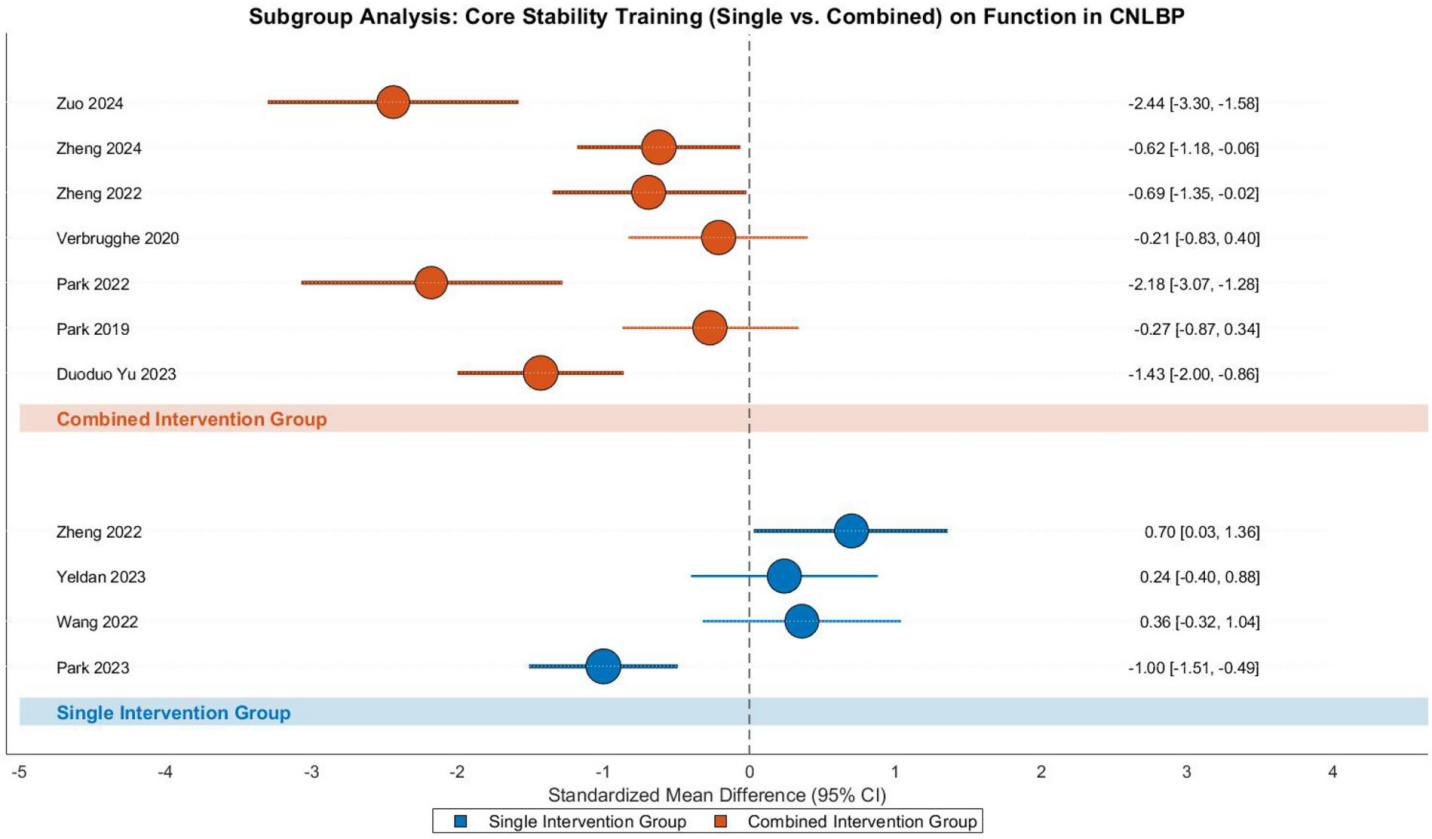
Subgroup meta-analysis forest plot of the functional improvement effect of core stability training on patients with CNLBP.

This meta-analysis evaluated the functional improvement effects of core stability training in CNLBP patients through subgroup analysis of 11 randomized controlled trials (4 single-modality studies and 7 combined-intervention studies), involving 479 participants (237 in intervention groups and 242 in controls). The analysis revealed substantial heterogeneity across studies (Tau^2^ = 0.65, *I*^2^ = 86%, *P* < 0.00001), necessitating the use of a random-effects model. The overall pooled effect size demonstrated statistically significant functional improvement with core stability training (SMD = −0.66, 95% CI: −1.18 to −0.14, *Z* = 2.49, *P* = 0.01), representing a moderate treatment effect according to Cohen's criteria (Figure 20).

Subgroup analysis showed contrasting results between intervention types. The single-modality subgroup (4 studies) showed no significant functional improvement (SMD = 0.06, 95% CI: −0.75 to 0.86, *Z* = 0.14, *P* = 0.89) with considerable heterogeneity (Tau^2^ = 0.57, *I*^2^ = 85%), where Zheng 2022 (SMD = 0.70) and Park 2023 (SMD = −1.00) demonstrated opposing effect directions, potentially due to variations in intervention intensity or outcome assessment tools. In contrast, the combined-intervention subgroup (7 studies) demonstrated strong effects (SMD = −1.07, 95% CI: −1.66 to −0.48, *Z* = 3.53, *P* = 0.0004) with substantial heterogeneity (Tau^2^ = 0.52, *I*^2^ = 82%), where Zuo 2024 (SMD = −2.44) and Park 2022 (SMD = −2.18) contributed most significantly to the effect size, possibly due to high-intensity combined interventions (e.g., integration with SET).

**Figure 21:**
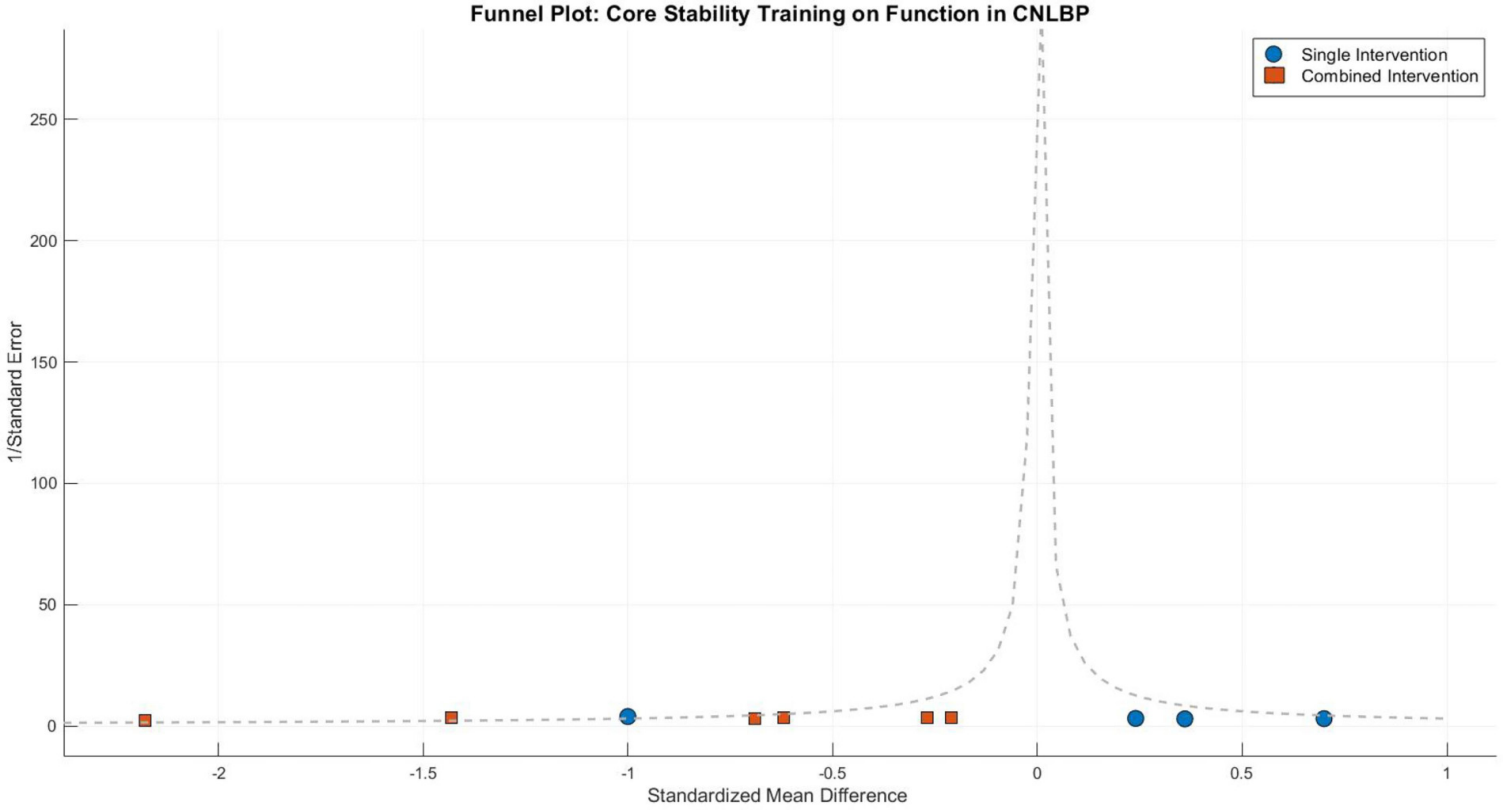
Funnel plot of the subgroup meta-analysis of the functional improvement effect of core stability training on patients with CNLBP.

Between-subgroup comparisons revealed statistically significant differences (Chi^2^ = 4.88, df = 1, *P* = 0.03, *I*^2^ = 79.5%), suggesting distinct mechanisms of functional improvement between single and combined intervention approaches. Funnel plot asymmetry demonstrated divergent patterns: the single-modality subgroup showed dispersed effect sizes (SMD = 0.06) with contradictory directions between studies (e.g., Zheng 2022 vs. Park 2023), reflecting high heterogeneity (*I*^2^ = 85%) likely due to intervention intensity or assessment tool variations; while the combined-intervention subgroup clustered in the strong-effect region (SMD = −1.07) despite persistent heterogeneity (*I*^2^ = 82%). These findings highlight the importance of intervention design in achieving functional improvements for CNLBP patients (Figure 21).

#### SET-function

**Figure 22:**
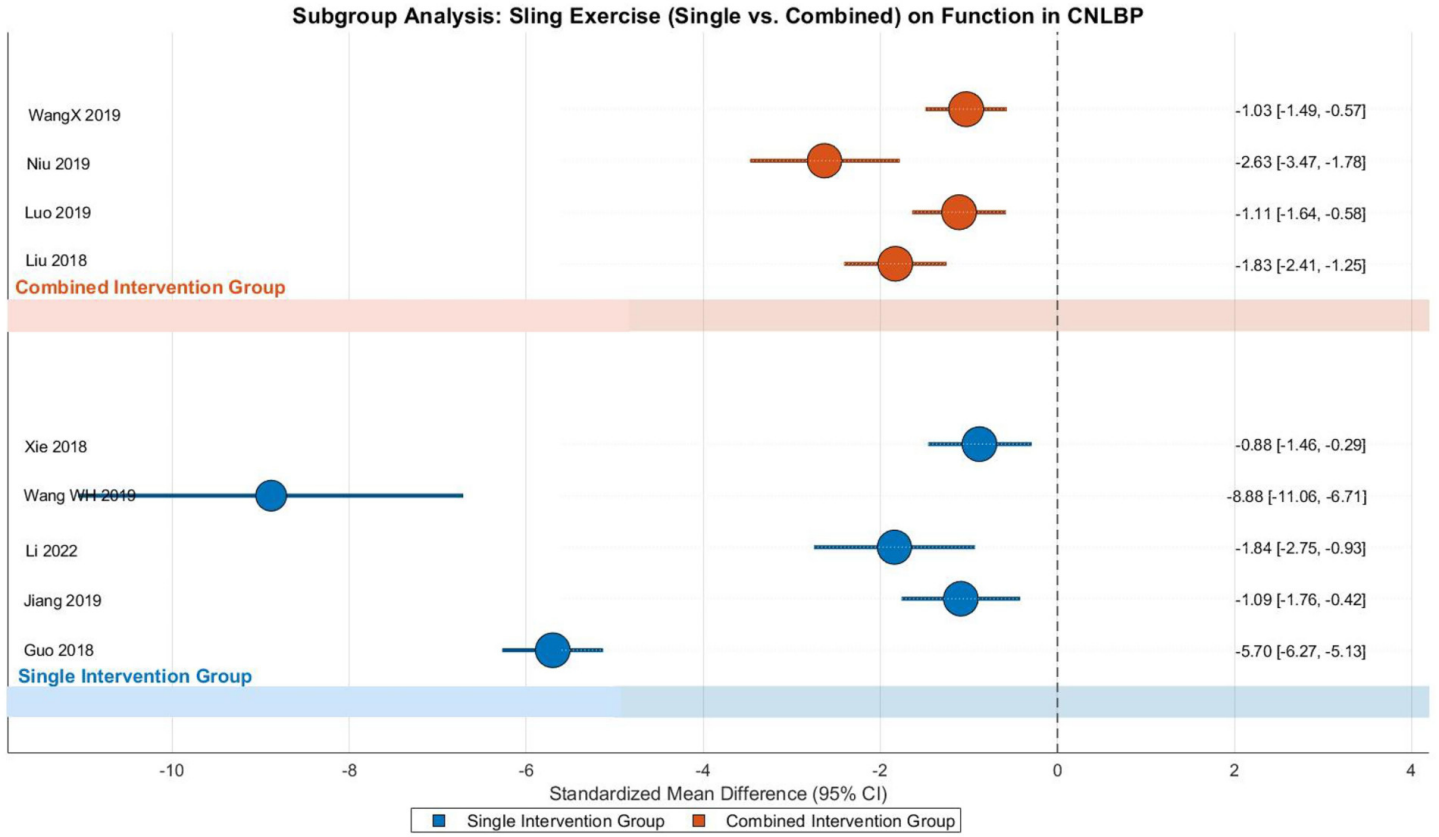
Subgroup meta-analysis forest plot of the functional improvement effect of SET on patients with CNLBP.

This meta-analysis investigated the effects of SET on functional improvement in CNLBP patients through subgroup analysis of 9 randomized controlled trials (5 single-modality studies and 4 combined-intervention studies), involving 655 participants (332 in intervention groups and 323 in controls). The analysis demonstrated extreme heterogeneity across studies (Tau^2^ = 3.29, *I*^2^ = 97%, *P* < 0.00001), justifying the application of a random-effects model. The pooled results revealed that SET yielded significantly greater functional improvement compared to control interventions (SMD = −2.60, 95% CI: −3.82 to −1.38, *Z* = 4.18, *P* < 0.0001), representing an exceptionally strong treatment effect according to Cohen's criteria (Figure 22).

Subgroup analysis showed that both intervention types produced substantial effects, though with notable differences in magnitude and variability. The single-modality subgroup (5 studies) demonstrated extremely strong effects (SMD = −3.54, 95% CI: −5.94 to −1.14, *Z* = 2.90, *P* = 0.004) with remarkable heterogeneity (Tau^2^ = 7.16, *I*^2^ = 98%), where Wang WH 2019 (SMD = −8.88) and Guo 2018 (SMD = −5.70) contributed extreme effect sizes, potentially due to large sample sizes (e.g., *N* = 120) or particularly intensive intervention protocols. The combined-intervention subgroup (4 studies) showed strong effects (SMD = −1.58, 95% CI: −2.21 to −0.96, *Z* = 4.96, *P* < 0.00001) with moderate-high heterogeneity (Tau^2^ = 0.31, *I*^2^ = 78%), where variations between studies like Niu 2019 (SMD = −2.63) and WangX 2019 (SMD = −1.03) suggested potential influences of different intervention combinations.

**Figure 23:**
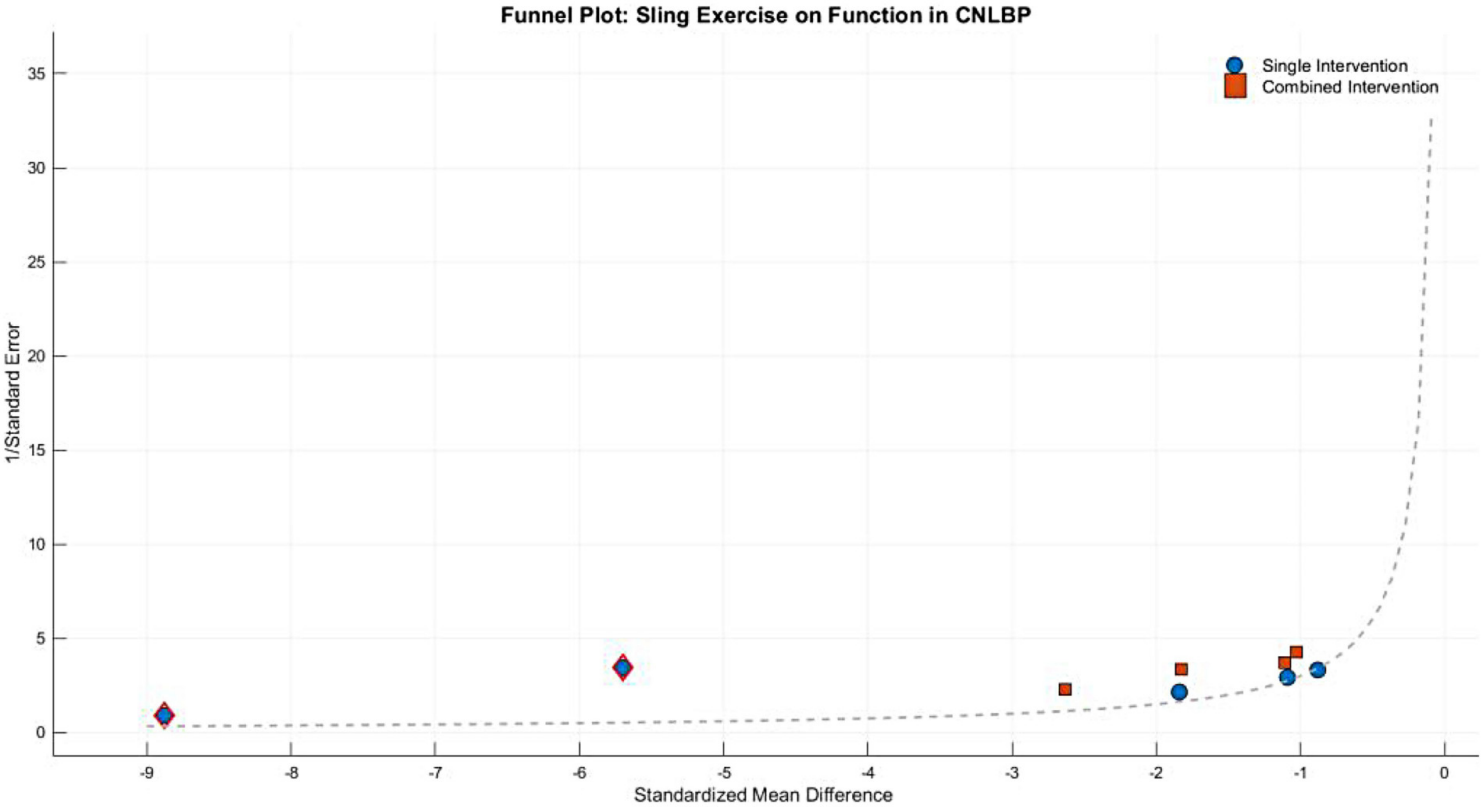
Funnel plot of the subgroup meta-analysis of the functional improvement effect of SET on patients with CNLBP.

Between-subgroup comparisons showed no statistically significant difference (Chi^2^ = 2.41, df = 1, *P* = 0.12, *I*^2^ = 58.4%), suggesting comparable effectiveness between single and combined intervention approaches. However, the extreme effect sizes and exceptionally high heterogeneity in the single-modality subgroup warrant cautious interpretation. Funnel plot asymmetry revealed substantial imbalance, with the single-modality subgroup showing extreme effect values (e.g., Wang WH 2019, SMD = −8.88) that dramatically inflated the overall effect estimate (SMD = −3.54) and contributed to extreme heterogeneity (*I*^2^ = 98%), while the combined-intervention subgroup demonstrated more moderate effects (SMD = −1.58) with relatively lower heterogeneity (*I*^2^ = 78%). These findings highlight both the potential efficacy of SET for functional improvement in CNLBP patients and the need for careful consideration of extreme outliers in the evidence base (Figure 23).

#### Pilates—Function

**Figure 24:**
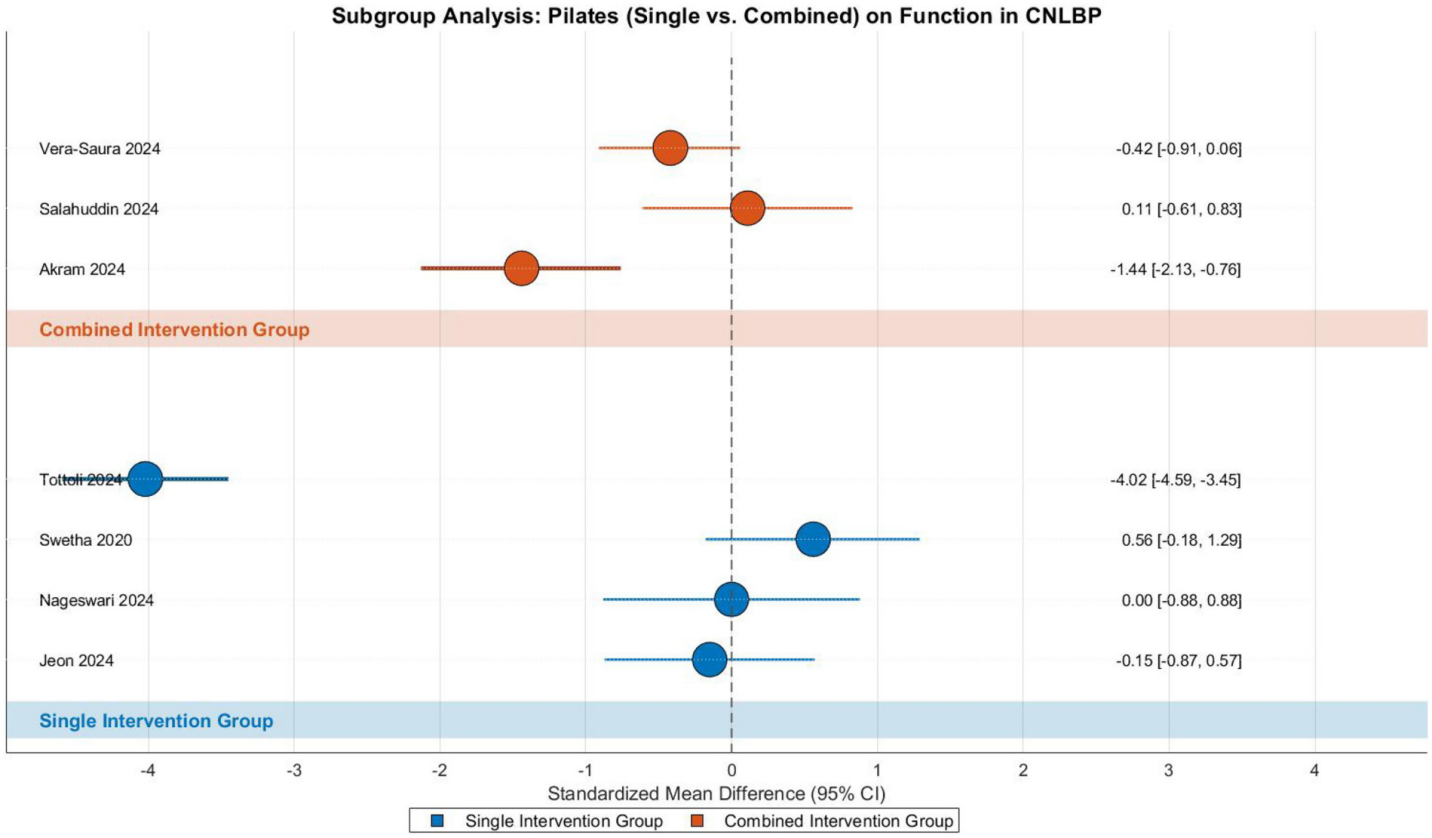
Subgroup meta-analysis forest plot of the functional improvement effect of Pilates on patients with CNLBP.

This meta-analysis evaluated the effects of Pilates training on functional improvement in CNLBP patients through subgroup analysis of 7 randomized controlled trials (4 single-modality studies and 3 combined-intervention studies), involving 364 participants (182 in each group). The analysis revealed extreme between-study heterogeneity (Tau^2^ = 2.74, *I*^2^ = 96%, *P* < 0.00001), necessitating the use of a random-effects model. The overall pooled effect size indicated no statistically significant functional improvement (SMD = −0.78, 95% CI: −2.03 to 0.48, *Z* = 1.21, *P* = 0.22), with the effect magnitude (|SMD| = 0.78) approaching but not reaching the threshold for a medium effect according to Cohen's criteria, while demonstrating insufficient result stability (Figure 24).

Subgroup analysis showed that neither intervention type achieved significant functional improvement. The single-modality subgroup (4 studies) demonstrated no significant effects (SMD = −0.91, 95% CI: −3.25 to 1.43, *Z* = 0.77, *P* = 0.44) with extreme heterogeneity (Tau^2^ = 5.56, *I*^2^ = 98%), where Tottoli 2024 (SMD = −4.02)—potentially due to its large sample size (*N* = 145) and high-intensity intervention—substantially lowered the effect estimate, while Swetha 2020 (SMD = 0.56) showed an opposite trend, possibly reflecting differences in assessment tools or intervention frequency. Similarly, the combined-intervention subgroup (3 studies) showed no significant improvement (SMD = −0.58, 95% CI: −1.39 to 0.22, *Z* = 1.42, *P* = 0.16) with considerable heterogeneity (Tau^2^ = 0.40, *I*^2^ = 80%), where Akram 2024 (SMD = −1.44) and Vera-Saura 2024 (SMD = −0.42) demonstrated consistent but varying effect directions. Notably, Merlo 2024 was excluded from analysis due to zero standard deviation in the control group. Between-subgroup comparisons revealed no significant difference (Chi^2^ = 0.07, df = 1, *P* = 0.79, *I*^2^ = 0%), suggesting comparable effects between intervention types.

**Figure 25:**
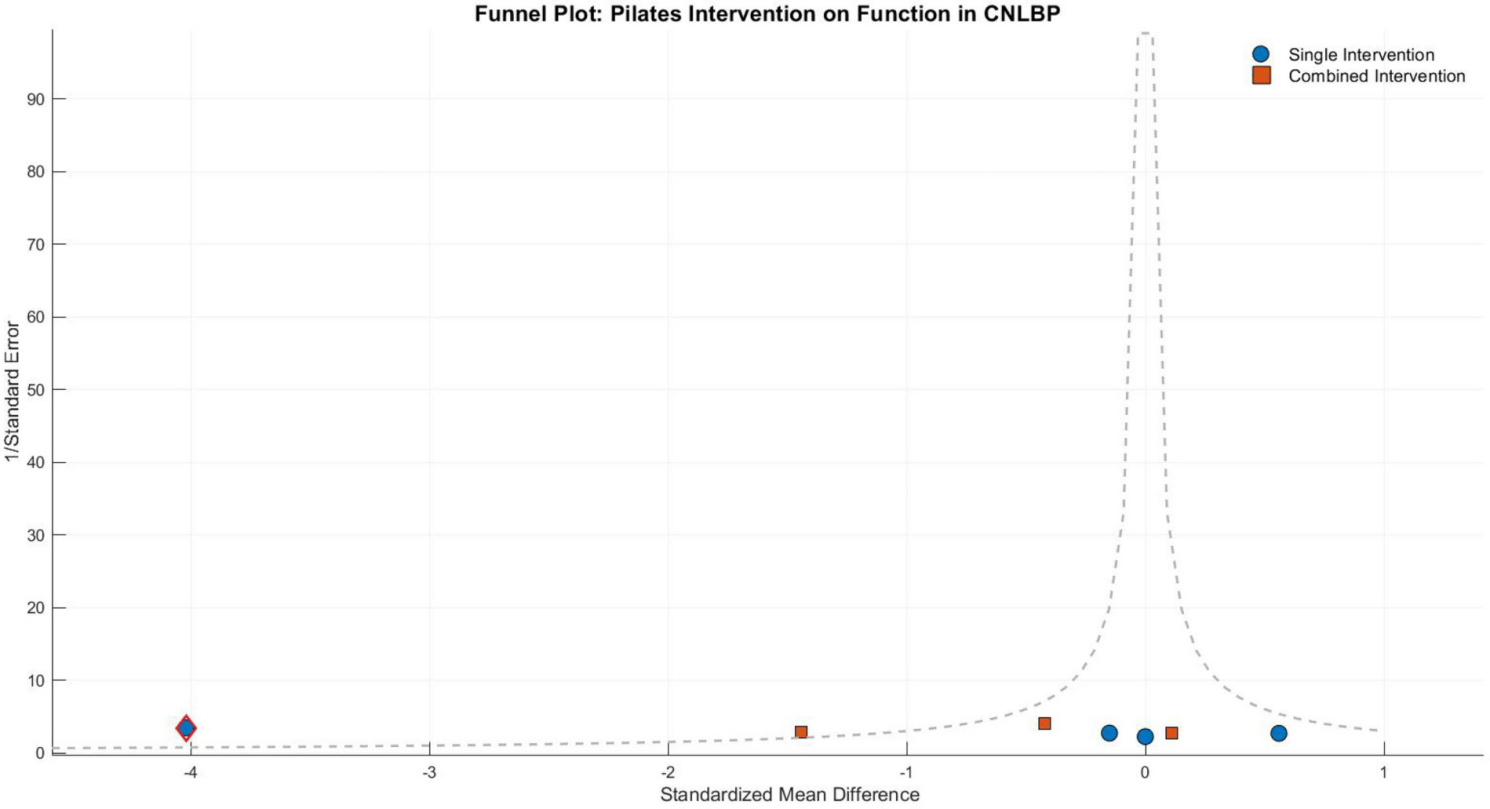
Funnel plot of the subgroup meta-analysis of the functional improvement effect of Pilates on patients with CNLBP.

The funnel plot displayed marked asymmetry, with the single-modality subgroup showing both extreme values (e.g., Tottoli 2024, SMD = −4.02) and counter-directional effects (e.g., Swetha 2020, SMD = 0.56) contributing to extreme heterogeneity (*I*^2^ = 98%), likely reflecting variations in intervention intensity or outcome measures. The combined-intervention subgroup showed more moderate but still substantial dispersion (SMD = −0.58, *I*^2^ = 80%), indicating persistent variability in treatment effects. These findings suggest that while Pilates training may show potential for functional improvement in CNLBP patients, the current evidence remains inconclusive due to extreme heterogeneity and inconsistent results across studies (Figure 25).

#### Breathing training—function

**Figure 26:**
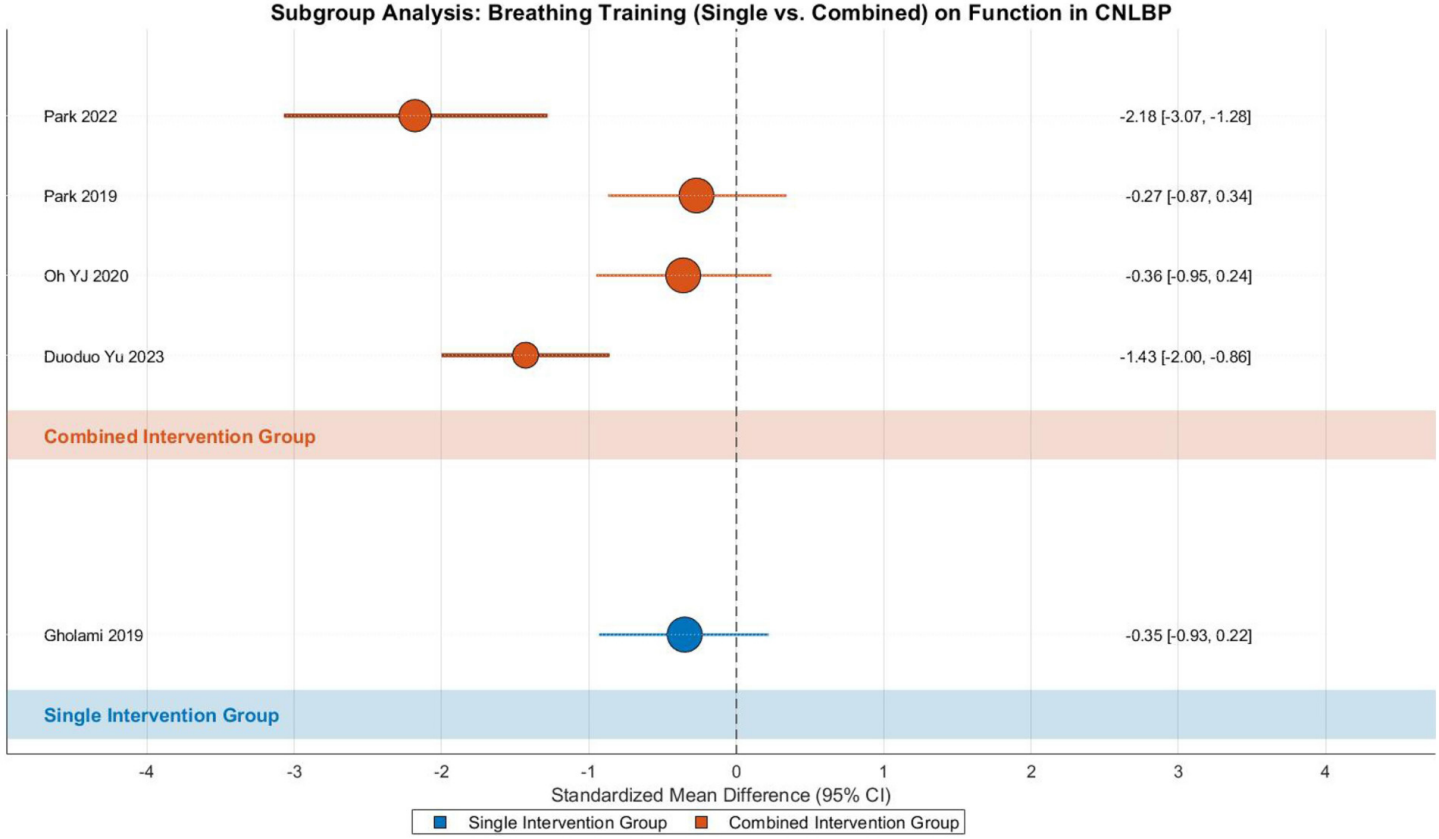
Subgroup meta-analysis forest plot of the functional improvement effect of respiratory training on patients with CNLBP.

This meta-analysis examined the effects of breathing exercises on functional improvement in CNLBP through subgroup analysis of 5 randomized controlled trials (1 single-modality study and 4 combined-intervention studies), involving 226 participants (112 in intervention groups and 114 in controls). The analysis demonstrated substantial heterogeneity across studies (Tau^2^ = 0.44, *I*^2^ = 81%, *P* = 0.0003), warranting the use of a random-effects model. The pooled results revealed that breathing exercises produced statistically significant functional improvement (SMD = −0.87, 95% CI: −1.52 to −0.22, *Z* = 2.62, *P* = 0.009), with the effect size approaching the threshold for a strong effect according to Cohen's criteria (|SMD| ≥ 0.8) (Figure 26).

Subgroup analysis revealed distinct patterns between intervention approaches. The single study in the standalone breathing exercise subgroup (Gholami 2019) showed no significant functional improvement (SMD = −0.35, 95% CI: −0.93 to 0.22, *Z* = 1.20, *P* = 0.23), representing a weak treatment effect. In contrast, the combined-intervention subgroup (4 studies) demonstrated strong effects (SMD = −1.02, 95% CI: −1.82 to −0.21, *Z* = 2.46, *P* = 0.01) despite considerable heterogeneity (Tau^2^ = 0.56, *I*^2^ = 84%). Within this subgroup, Park 2022 (SMD = −2.18) and Duoduo Yu 2023 (SMD = −1.43) contributed substantially to the overall effect, potentially due to higher-intensity combined interventions (e.g., integration with core stability training), while Oh YJ 2020 (SMD = −0.36) and Park 2019 (SMD = −0.27) showed more modest effects, possibly reflecting differences in adjunct therapy intensity.

**Figure 27:**
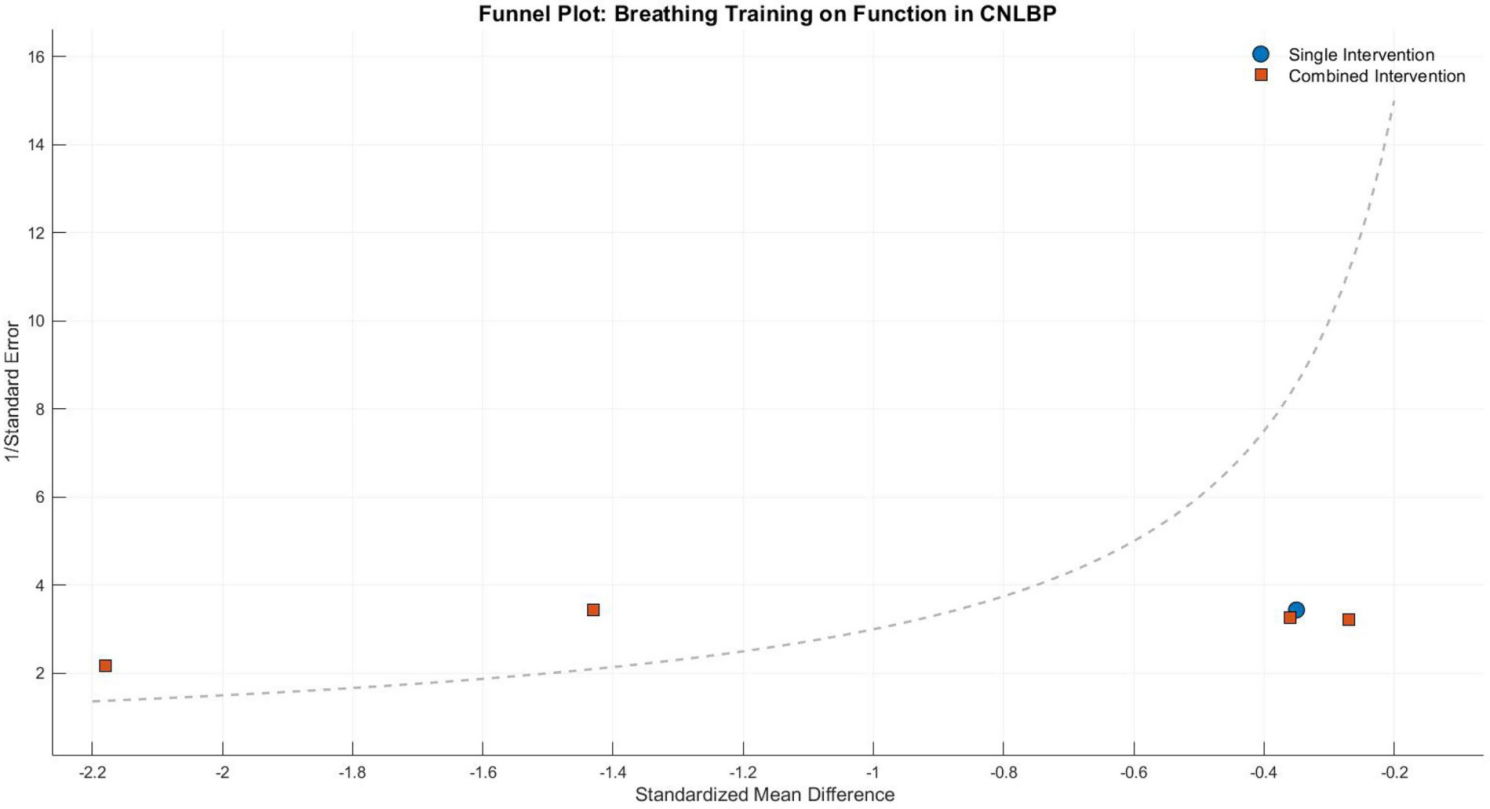
Forest plot of the subgroup meta-analysis of the functional improvement effect of respiratory training on patients with CNLBP.

Between-subgroup comparisons showed no statistically significant difference (Chi^2^ = 1.72, df = 1, *P* = 0.19, *I*^2^ = 41.7%), suggesting comparable effects between intervention types, though the more pronounced effects in the combined-intervention subgroup warrant consideration. Funnel plot asymmetry revealed that while the combined-intervention subgroup showed dispersed effect sizes (SMD = −1.02, *I*^2^ = 84%) with extreme values (e.g., Park 2022, SMD = −2.18) reflecting variability in adjunct therapy intensity, the single study in the standalone subgroup (SMD = −0.35) provided insufficient evidence for reliable conclusions. These findings suggest that while breathing exercises, particularly when combined with other therapies, may improve function in CNLBP patients, the limited number of studies and substantial heterogeneity necessitate cautious interpretation of these results (Figure 27).

### Single-Modality versus combined interventions: subgroup summary

To provide a clear overview of the comparative efficacy between single-modality and combined interventions across all training types, we have summarized the subgroup findings in Table 3.

**Table 3:** Summary of subgroup analysis: single-modality vs. combined intervention.

Training type	Subgroup	No. of studies	Pain (SMD,95% CI)	Function (SMD, 95% CI)
Core stability training	Single-modality	11	−0.36 [−0.91, 0.19]	0.06 [−0.75,0.86]
	Combined	1	−0.73 [−1.40,−0.06]	−1.07 [−1.66,−0.48]
SET	Single-modality	5	−1.74 [−2.52,−0.96]	−3.54 [−5.94,−1.14]
	Combined	4	−1.10 [−1.46,−0.73]	−1.58 [−2.21,−0.96]
Pilates	Single-modality	3	−2.42 [−4.86, 0.02]	−0.91[−3.25, 1.43]
	Combined	5	−0.92[−1.62,−0.21]	−0.58 [−1.39,0.22]
Breathing training	Single-modality	2(pain)/1(function)	−1.40 [−1.85,−0.94]	−0.35 [−0.93,0.22]
	Combined	5(pain)/4(function)	−0.50 [−1.28,0.29]	−1.02 [−1.82,−0.21]

These results demonstrate that the comparative efficacy of single-modality vs. combined approaches varies by training type and outcome measure. Notably, for breathing training, single-modality interventions showed consistent strong effects for pain relief (*I*^2^ = 0%), while combined interventions yielded non-significant results with high heterogeneity.

### Effects of different intervention cycles

#### The efficacy of <8 weeks of different core training on patients' pain and function

##### Pain

**Figure 28:**
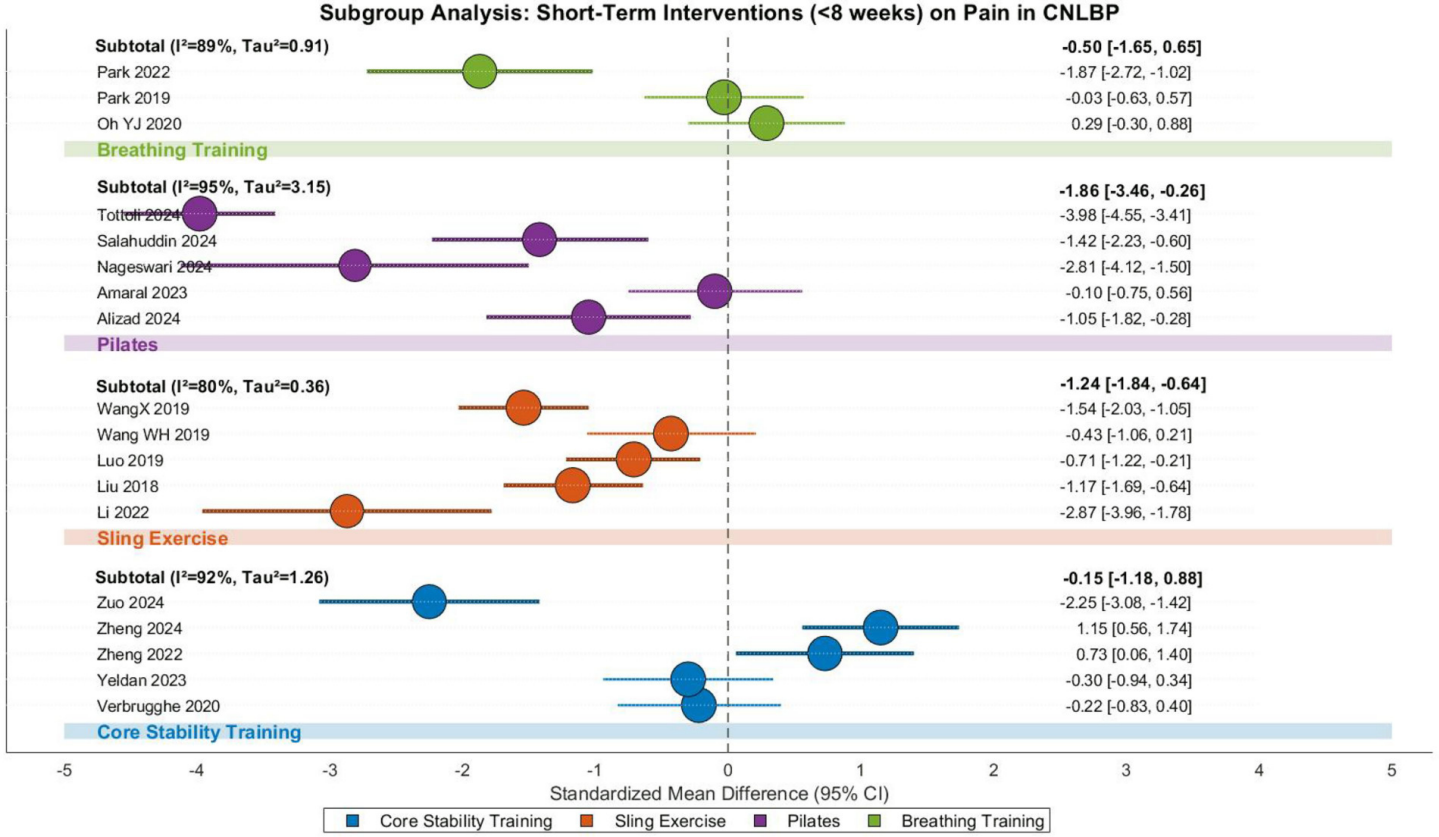
Subgroup meta-analysis forest plot of the effect of different core stability training <8 weeks on pain improvement in patients with CNLBP.

This meta-analysis evaluated the efficacy of short-term (<8 weeks) core training interventions for pain relief in CNLBP patients, incorporating 18 randomized controlled trials with 869 participants (435 in intervention groups and 434 in controls). The analysis revealed extreme between-study heterogeneity (Tau^2^ = 1.62, *I*^2^ = 94%, *P* < 0.00001), necessitating a random-effects model approach. The pooled results demonstrated that short-term core training significantly reduced pain compared to control interventions (SMD = −1.00, 95% CI: −1.61 to −0.39, *Z* = 3.20, *P* = 0.001), representing a strong treatment effect according to Cohen's criteria (Figure 28).

Subgroup analyses revealed distinct patterns across intervention modalities. Conventional core stability training (5 studies) showed no significant pain reduction (SMD = −0.15, 95% CI: −1.18 to 0.88, *Z* = 0.29, *P* = 0.77) with extreme heterogeneity (*I*^2^ = 92%), where contradictory findings between Zuo 2024 (SMD = −2.25) and Zheng 2024 (SMD = 1.15) may reflect variations in training intensity or outcome measures. Suspension training (5 studies) demonstrated strong effects (SMD = −1.24, 95% CI: −1.84 to −0.64, *Z* = 4.06, *P* < 0.0001, *I*^2^ = 80%), with Li 2022 (SMD = −2.87) and WangX 2019 (SMD = −1.54) showing particularly robust outcomes potentially attributable to higher training frequency. Pilates interventions (5 studies) yielded the strongest effects (SMD = −1.86, 95% CI: −3.46 to −0.26, *Z* = 2.28, *P* = 0.02) despite extreme heterogeneity (*I*^2^ = 95%), where Tottoli 2024's intensive protocol (SMD = −3.98) contrasted with Amaral 2023's modest results (SMD = −0.10). Breathing exercises (3 studies) showed non-significant effects (SMD = −0.50, 95% CI: −1.65 to 0.65, *Z* = 0.85, *P* = 0.40, *I*^2^ = 89%), with divergent outcomes between Park 2022 (SMD = −1.87) and Oh YJ 2020 (SMD = 0.29) possibly reflecting differences in adjunct core training intensity.

**Figure 29:**
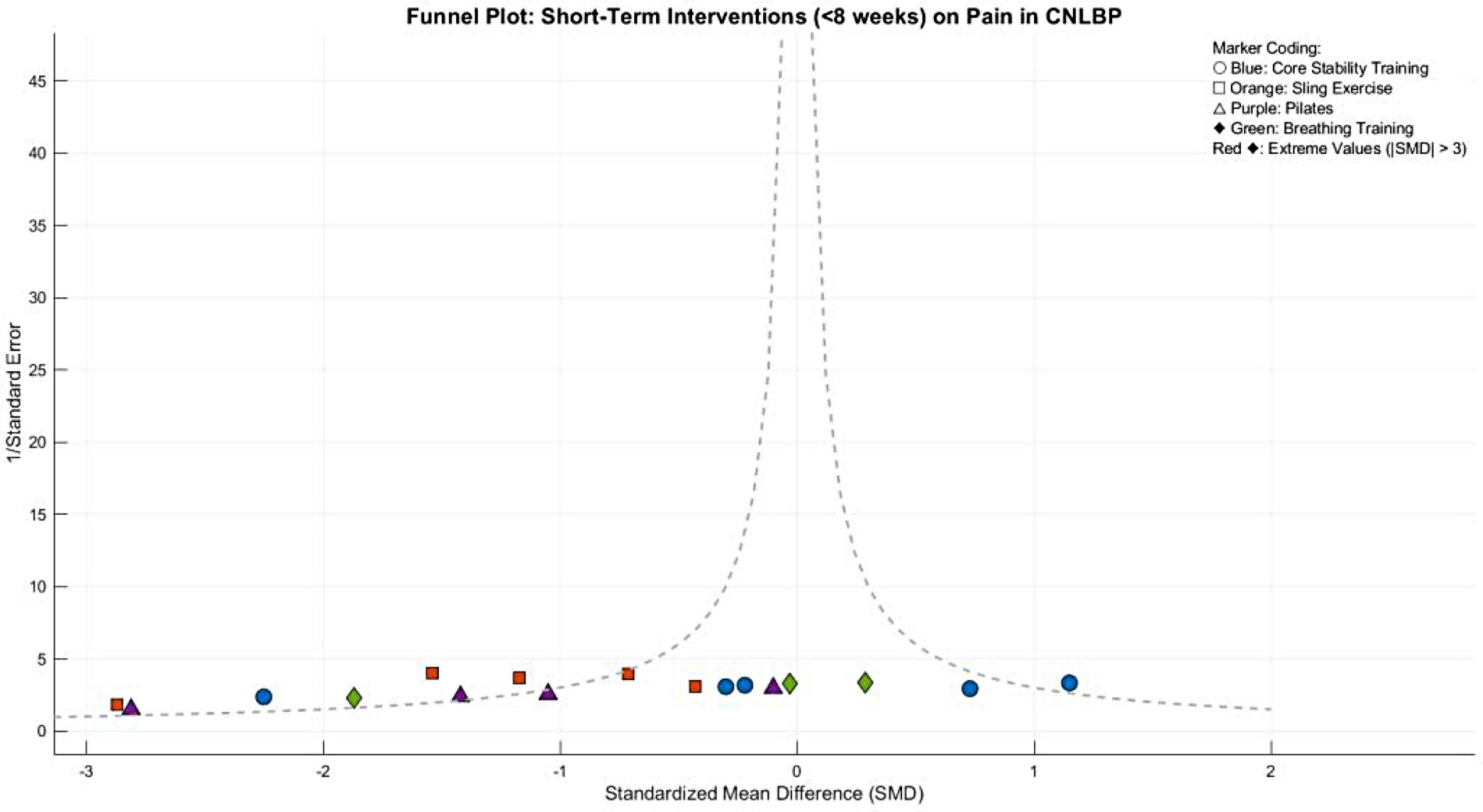
Funnel plot of the subgroup meta-analysis of the pain improvement effect of different core stability training <8 weeks on patients with CNLBP.

Between-subgroup comparisons revealed no statistically significant differences (Chi^2^ = 5.02, df = 3, *P* = 0.17, *I*^2^ = 40.2%), suggesting comparable pain-relieving effects across intervention types, though the particularly strong effects of Pilates and suspension training merit clinical consideration. Funnel plot asymmetryindicated potential publication bias, with smaller studies showing widely dispersed effect sizes and extreme outliers (e.g., Tottoli 2024's SMD = −3.98 in Pilates) disproportionately influencing overall estimates, while larger studies clustered in the strong-effect ranges for suspension training and Pilates (SMD = −1.86). These findings suggest that while short-term core training generally benefits CNLBP patients, the optimal modality may depend on specific clinical contexts and implementation parameters(SMD = −1.24) (Figure 29).

##### Function

**Figure 30:**
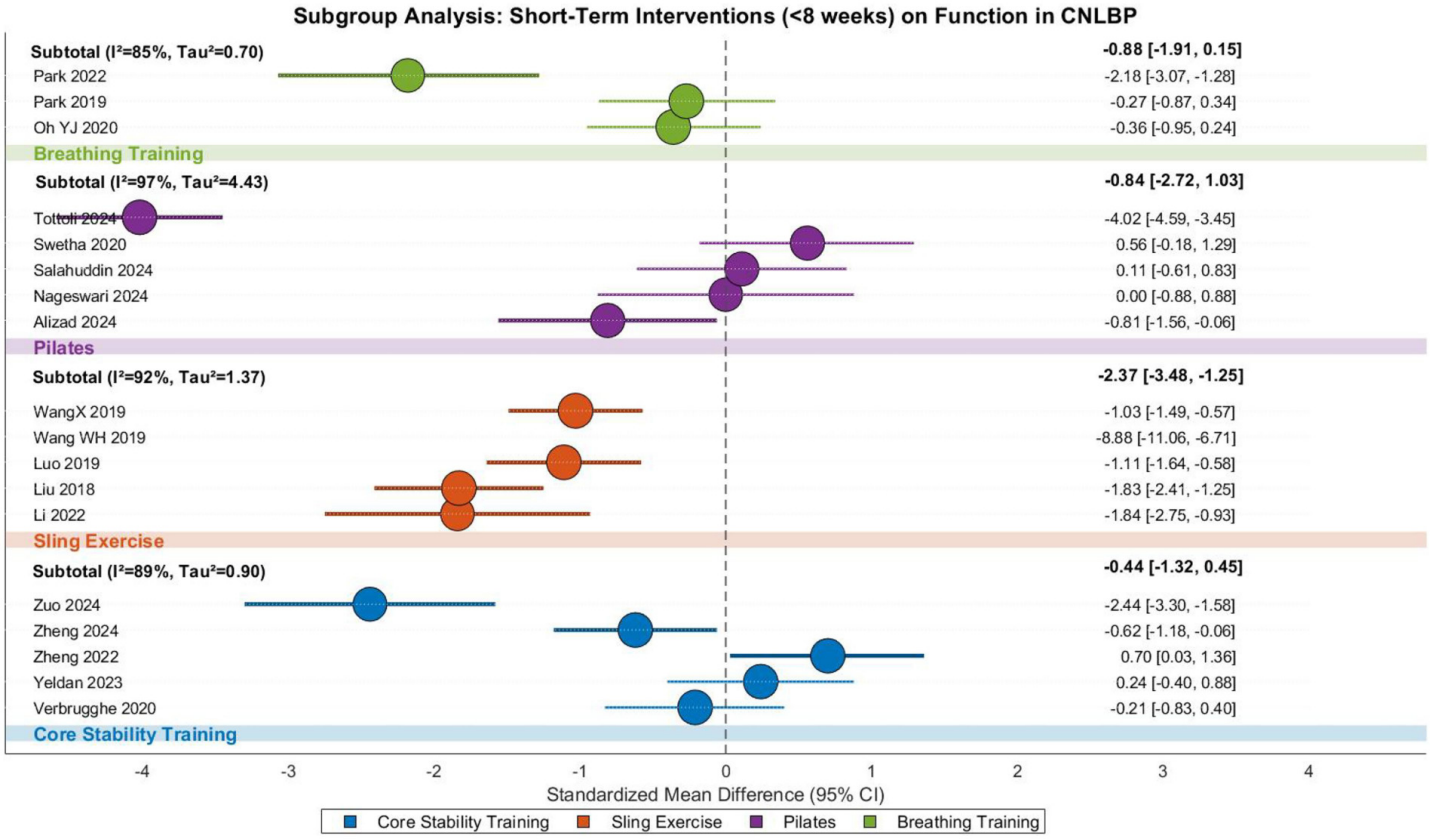
Subgroup meta-analysis forest plot of the functional improvement effect of different core stability training <8 weeks on patients with CNLBP.

This meta-analysis investigated the functional improvement effects of short-term (<8 weeks) core training in CNLBP patients, including 17 randomized controlled trials with 863 participants (433 in intervention groups and 430 in controls).The analysis demonstrated extreme between-study heterogeneity (Tau^2^ = 1.74, *I*^2^ = 94%, *P* < 0.00001), requiring a random-effects model approach. The pooled results indicated that short-term core training significantly improved functional outcomes compared to control interventions (SMD = −1.17, 95% CI: −1.81 to −0.53, *Z* = 3.60, *P* = 0.0003), representing a strong treatment effect according to Cohen's criteria (Figure 30).

Subgroup analyses revealed substantial variation across intervention types. Conventional core stability training (5 studies) showed no significant functional improvement (SMD = −0.44, 95% CI: −1.32 to 0.45, *Z* = 0.97, *P* = 0.33) with high heterogeneity (*I*^2^ = 89%), where contradictory findings between Zuo 2024 (SMD = −2.44) and Zheng 2022 (SMD = 0.70) may reflect differences in intervention protocols or outcome measures. SET (, 5 studies) demonstrated exceptionally strong effects (SMD = −2.37, 95% CI: −3.48 to −1.25, *Z* = 4.16, *P* < 0.0001) despite extreme heterogeneity (*I*^2^ = 92%), with Wang WH 2019's extreme effect (SMD = −8.88) disproportionately influencing results, while Li 2022 (SMD = −1.84) and Liu 2018 (SMD = −1.83) showed consistent but varying effect sizes potentially related to training frequency or adjunct therapies.

Pilates interventions (5 studies) showed non-significant effects (SMD = −0.84, 95% CI: −2.72 to 1.03, *Z* = 0.88, *P* = 0.38) with extreme heterogeneity (*I*^2^ = 97%), where Tottoli 2024's intensive protocol (SMD = −4.02) contrasted with neutral (Nageswari 2024, SMD = 0.00) and counter-directional (Swetha 2020, SMD = 0.56) findings. Breathing exercises (3 studies) also showed non-significant improvement (SMD = −0.88, 95% CI: −1.91 to 0.15, *Z* = 1.67, *P* = 0.10, *I*^2^ = 85%), with marked variation between Park 2022 (SMD = −2.18) and Oh YJ 2020 (SMD = −0.36).

Between-subgroup comparisons approached statistical significance (Chi^2^ = 7.36, df = 3, *P* = 0.06, *I*^2^ = 59.2%), suggesting that suspension training may offer superior functional benefits compared to other modalities in short-term interventions. While SET demonstrated particularly strong effects (SMD = −2.37), the extreme values (e.g., Wang WH 2019) and substantial heterogeneity (*I*^2^ = 92%) necessitate cautious interpretation. Core stability training, Pilates, and breathing exercises collectively failed to show significant functional improvement in this short-term context.

**Figure 31:**
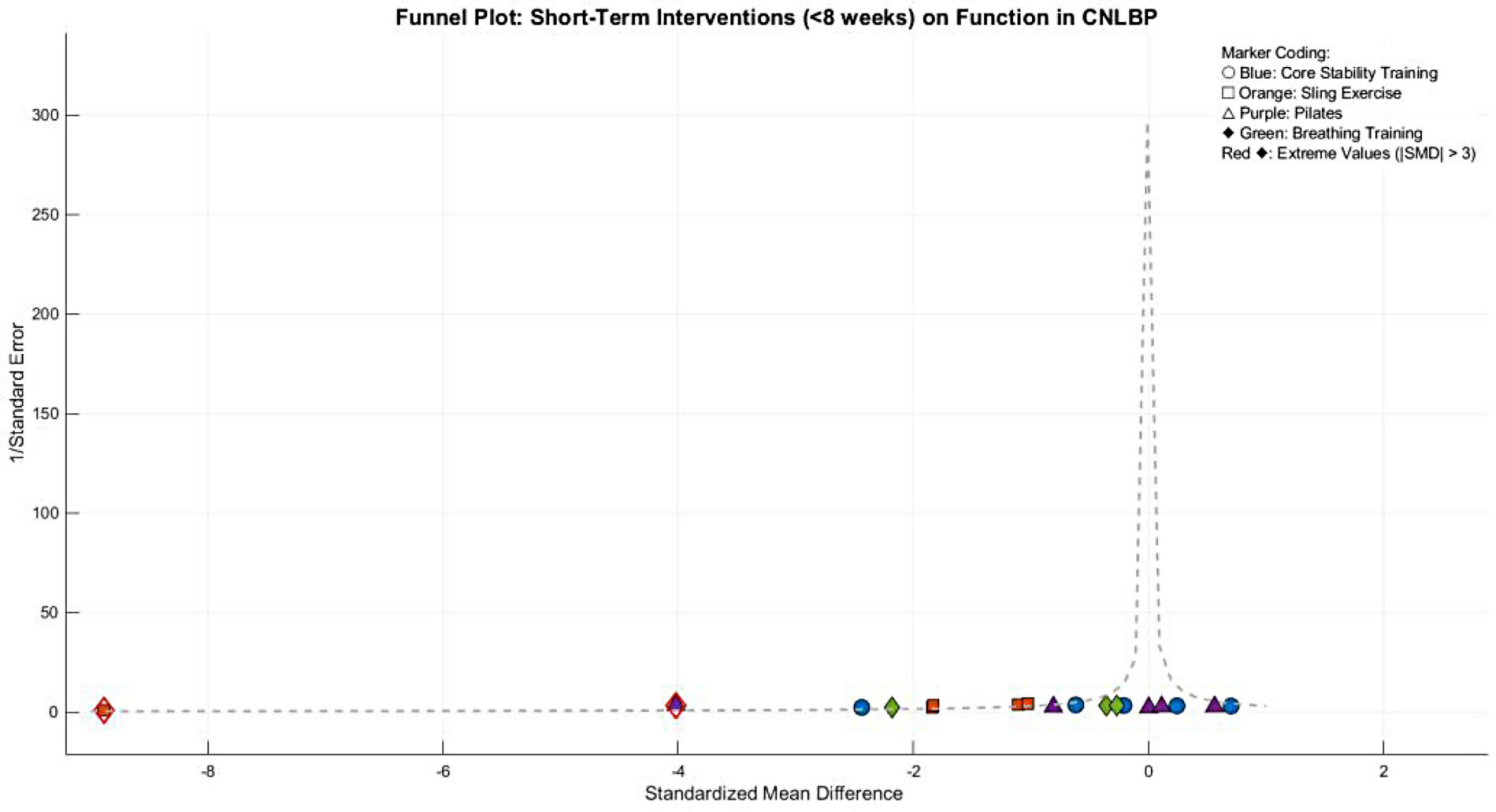
Funnel plot of the subgroup meta-analysis of the functional improvement effect of different core stability training <8 weeks on patients with CNLBP.

Funnel plot asymmetry revealed substantial imbalance, with suspension training studies (orange) contributing extreme effect sizes (e.g., Wang WH 2019, SMD = −8.88) that dramatically lowered the pooled estimate (SMD = −2.37), while Pilates (purple) and breathing exercise (green) studies showed dispersed and generally weaker effects. The contradictory distribution of core stability training studies (blue) reflected substantial heterogeneity (overall *I*^2^ = 94%) likely stemming from variations in intervention parameters (Figure 31).

#### The efficacy of ≥8 weeks of different core training on patients' pain and function

##### Pain

**Figure 32:**
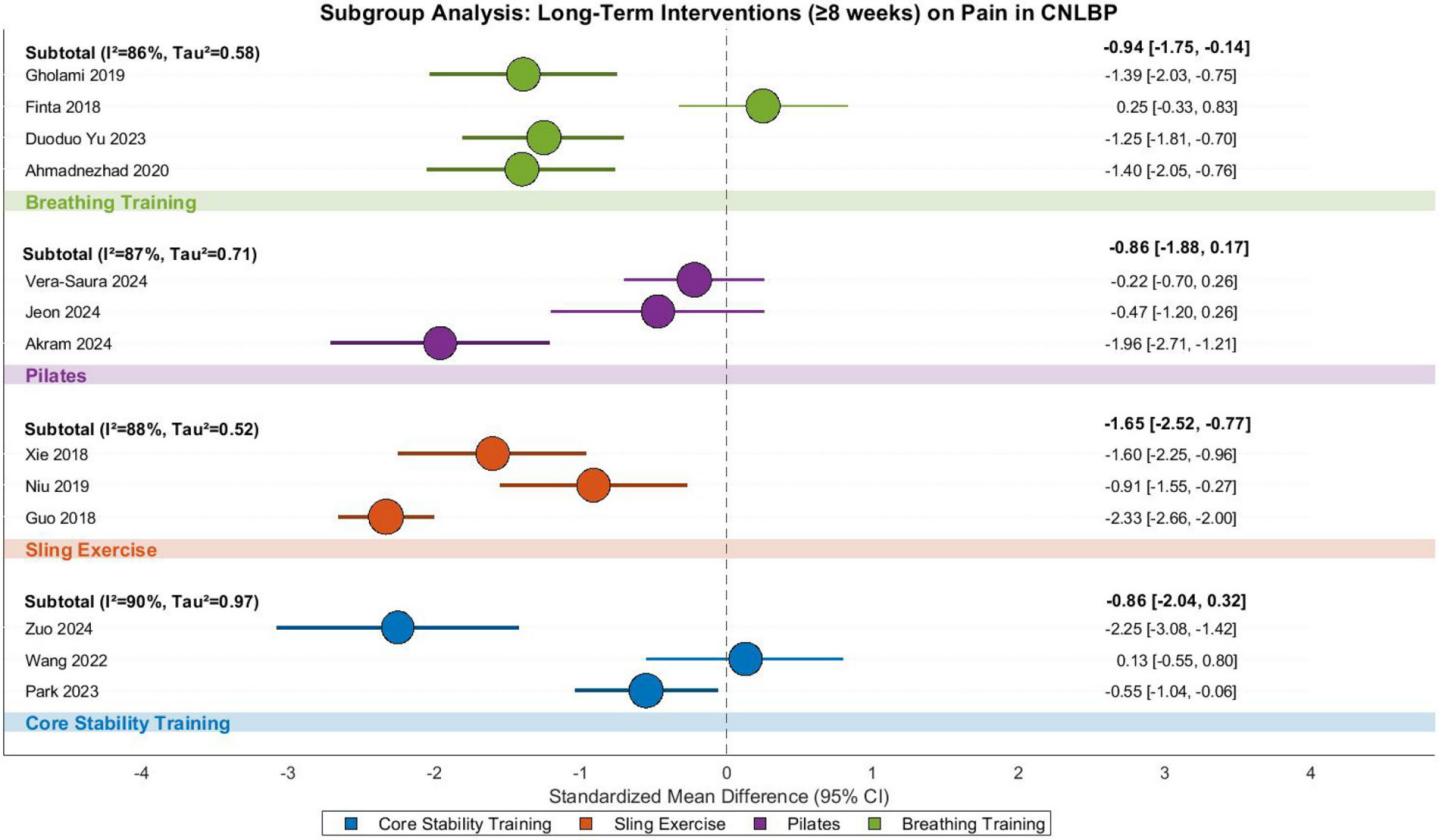
Subgroup meta-analysis forest plot of the improvement effect of different core stability training for ≥8 weeks on pain in patients with CNLBP.

This meta-analysis examined the pain-relieving effects of long-duration (≥8 weeks) core training in CNLBP patients, incorporating 13 randomized controlled trials with 849 participants (427 in intervention groups and 422 in controls). The analysis revealed substantial between-study heterogeneity (Tau^2^ = 0.80, *I*^2^ = 90%, *P* < 0.00001), necessitating a random-effects model. The pooled results demonstrated that extended core training significantly reduced pain compared to control interventions (SMD = −1.07, 95% CI: −1.58 to −0.55, *Z* = 4.07, *P* < 0.0001), representing a strong treatment effect according to Cohen's criteria (Figure 32).

Subgroup analyses showed differential effectiveness across modalities. Conventional core stability training (3 studies) showed non-significant pain reduction (SMD = −0.86, 95% CI: −2.04 to 0.32, *Z* = 1.43, *P* = 0.15) with extreme heterogeneity (*I*^2^ = 90%), where Zuo 2024 (SMD = −2.25) and Wang 2022 (SMD = 0.13) demonstrated opposing effects. Suspension exercise therapy (SET, 3 studies) yielded strong effects (SMD = −1.65, 95% CI: −2.52 to −0.77, *Z* = 3.68, *P* = 0.0002, *I*^2^ = 88%), with Guo 2018 (SMD = −2.33) and Xie 2018 (SMD = −1.60) showing particularly robust outcomes potentially attributable to higher training frequency.

Pilates interventions (4 studies) showed non-significant improvement (SMD = −0.86, 95% CI: −1.88 to 0.17, *Z* = 1.64, *P* = 0.10, *I*^2^ = 87%), with marked variation between Akram 2024 (SMD = −1.96) and Vera-Saura 2024 (SMD = −0.22). Breathing exercises (4 studies) demonstrated significant pain reduction (SMD = −0.94, 95% CI: −1.75 to −0.14, *Z* = 2.30, *P* = 0.02, *I*^2^ = 86%), where consistent effects in Ahmadnezhad (2020) and Gholami (2019) (SMD ≈ −1.40) contrasted with Finta 2018's counterintuitive finding (SMD = 0.25), possibly reflecting differences in adjunct exercise intensity.

**Figure 33:**
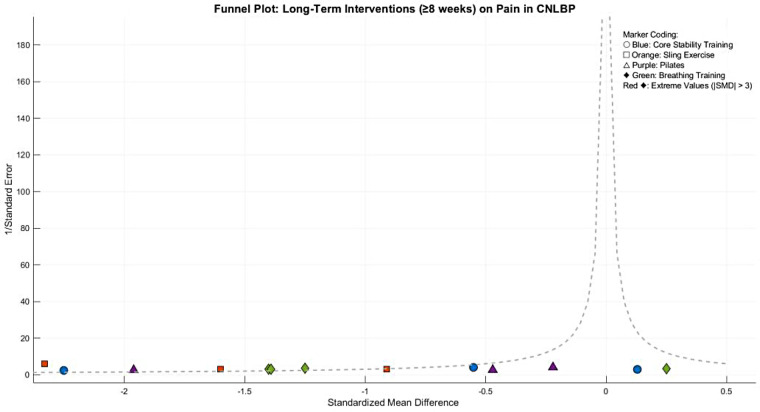
Funnel plot of the subgroup meta-analysis of the improvement effect of different core stability training for ≥8 weeks on pain in patients with CNLBP.

Between-subgroup comparisons showed no statistically significant differences (Chi^2^ = 2.01, df = 3, *P* = 0.57, *I*^2^ = 0%), suggesting comparable pain-relieving effects across intervention types, though the significant benefits of SET and breathing exercises merit clinical consideration. Funnel plot asymmetry revealed that SET (orange) and breathing exercise (green) studies clustered in the strong-effect region (SMD = −1.65/−0.94), while core stability (blue) and Pilates (purple) studies showed dispersed distributions, with some extreme values (e.g., Zuo 2024's SMD = −2.25 in core stability) reflecting substantial heterogeneity (overall *I*^2^ = 90%) likely stemming from intervention protocol variations. These findings suggest that while extended core training generally benefits CNLBP patients, the choice of specific modality should consider both efficacy evidence and practical implementation factors (Figure 33).

##### Function

**Figure 34:**
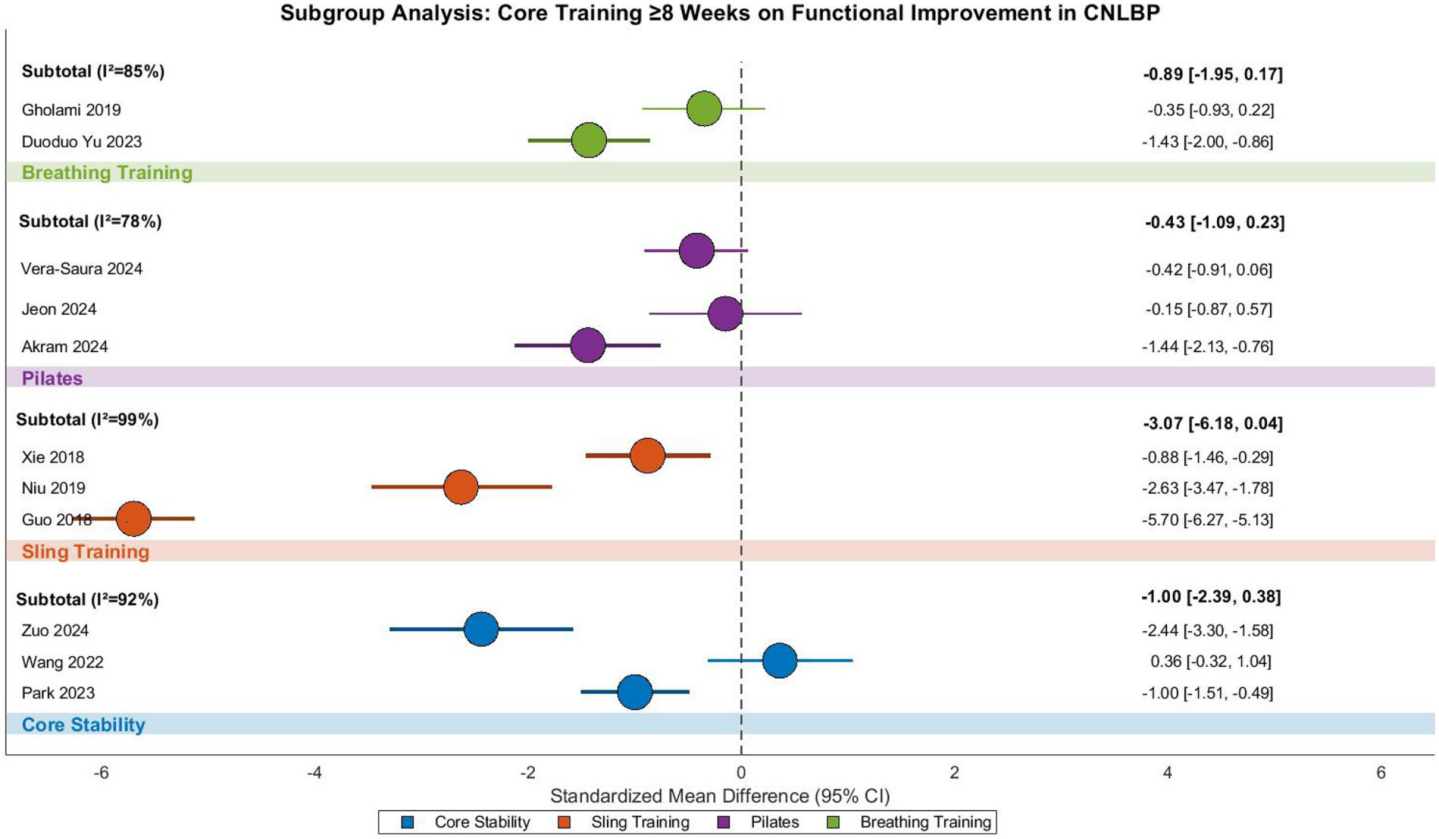
Subgroup meta-analysis forest plot of the functional improvement effect of different core stability training for ≥8 weeks on patients with CNLBP.

This meta-analysis evaluated the functional improvement effects of long-duration (≥8 weeks) core training in CNLBP patients, incorporating 12 randomized controlled trials with 755 participants (378 in intervention groups and 377 in controls). The analysis demonstrated extreme between-study heterogeneity (Tau^2^ = 2.85, *I*^2^ = 97%, *P* < 0.00001), requiring a random-effects model approach. The pooled results indicated that extended core training significantly improved functional outcomes compared to control interventions (SMD = −1.31, 95% CI: −2.29 to −0.34, *Z* = 2.64, *P* = 0.008), representing a strong treatment effect according to Cohen's criteria (Figure 34).

Subgroup analyses revealed substantial variation across intervention modalities. Conventional core stability training (3 studies) showed non-significant functional improvement (SMD = −1.00, 95% CI: −2.39 to 0.38, *Z* = 1.42, *P* = 0.16) with extreme heterogeneity (*I*^2^ = 92%), where Zuo 2024's dynamic training protocol (SMD = −2.44) contrasted sharply with Wang 2022's static approach (SMD = 0.36), potentially reflecting fundamental differences in intervention design or outcome assessment. Suspension exercise therapy (SET, 3 studies) demonstrated borderline significant effects (SMD = −3.07, 95% CI: −6.18 to 0.04, *Z* = 1.94, *P* = 0.05) with extraordinary heterogeneity (*I*^2^ = 99%), where Guo 2018 (SMD = −5.70) and Niu 2019 (SMD = −2.63) contributed extreme effect sizes that substantially influenced the pooled estimate.

Pilates interventions (4 studies) showed non-significant effects (SMD = −0.43, 95% CI: −1.09 to 0.23, *Z* = 1.27, *P* = 0.21) with considerable heterogeneity (*I*^2^ = 78%), where Akram 2024 (SMD = −1.44) and Vera-Saura 2024 (SMD = −0.42) demonstrated consistent but varying effect directions. Breathing exercises (2 studies) also showed non-significant improvement (SMD = −0.89, 95% CI: −1.95 to 0.17, *Z* = 1.65, *P* = 0.10, *I*^2^ = 85%), with marked variation between Duoduo Yu 2023 (SMD = −1.43) and Gholami 2019 (SMD = −0.35).

Between-subgroup comparisons revealed no statistically significant differences (Chi^2^ = 3.18, df = 3, *P* = 0.37, *I*^2^ = 5.6%), suggesting comparable functional benefits across intervention types. While SET showed the strongest point estimate (SMD = −3.07), the extreme values (e.g., Guo 2018) and extraordinary heterogeneity (*I*^2^ = 99%) raise concerns about result reliability. The non-significant findings for core stability training, Pilates, and breathing exercises may reflect intervention protocol variability (e.g., adjunct therapies, training frequency) and limited sample sizes.

**Figure 35:**
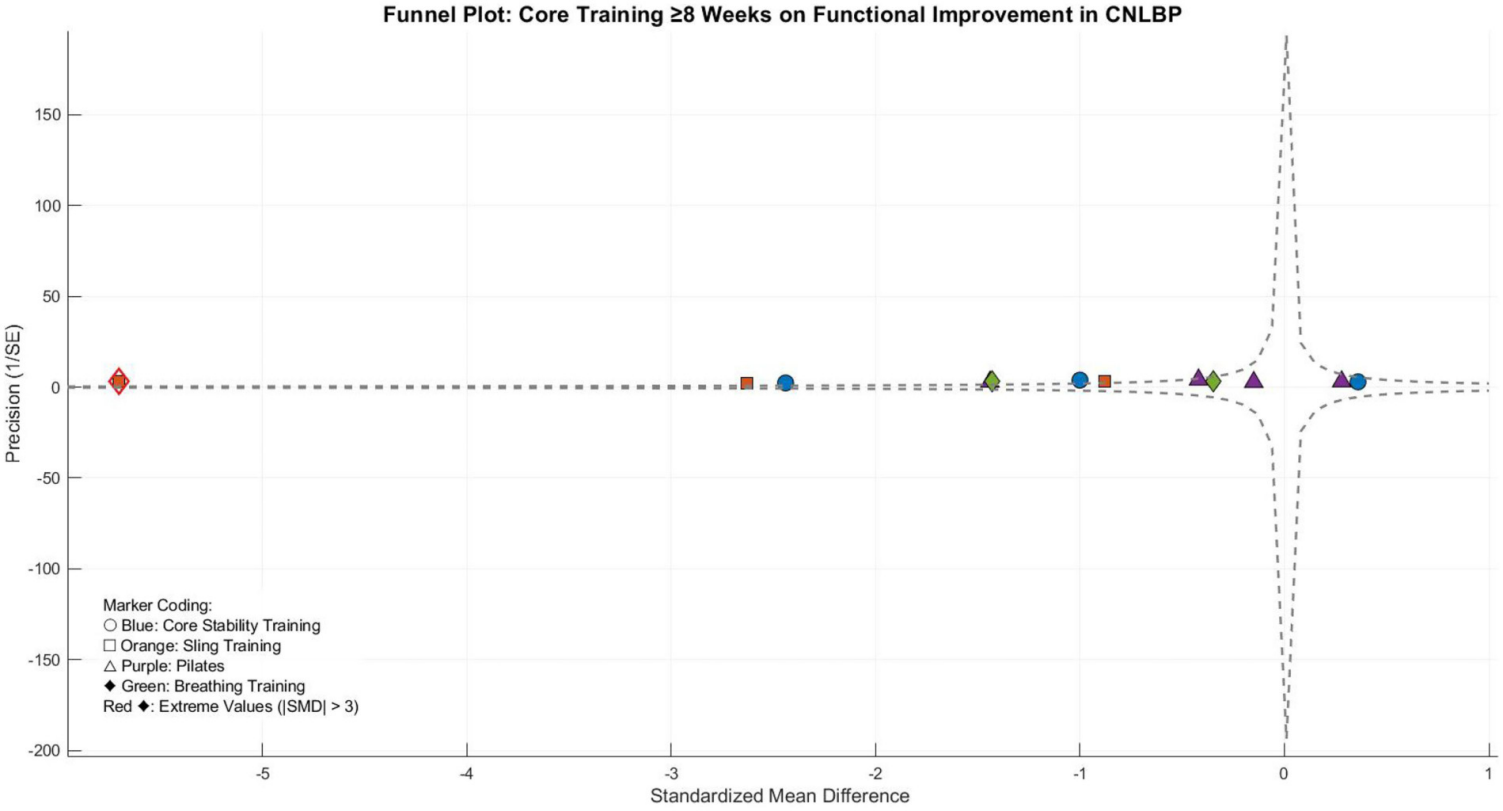
Funnel plot of the subgroup meta-analysis of the functional improvement effect of different core stability training for ≥8 weeks on patients with CNLBP.

Funnel plot asymmetry demonstrated substantial imbalance, with SET studies (orange) contributing extreme effect sizes (e.g., Guo 2018, SMD = −5.70) that dramatically lowered the pooled estimate (SMD = −3.07) and drove near-maximal heterogeneity (*I*^2^ = 99%). Other subgroups (core stability, Pilates, breathing exercises) showed dispersed and non-significant effects, reflecting intervention protocol differences and inconsistent outcome measures (Figure 35).

## Discussion

### Summary of principal findings

This meta-analysis provides robust evidence supporting core stability training for CNLBP management, demonstrating significant improvements in pain relief (SMD = −0.95) and functional recovery (SMD = −1.09). However, substantial heterogeneity (*I*^2^ = 93%–97%) necessitates personalized treatment approaches based on patient characteristics. Therapeutic benefits derive primarily from targeted activation of deep core musculature (transversus abdominis, multifidus) and subsequent neuromuscular adaptations, which reduce abnormal lumbar stress distribution and disrupt the pain-instability-compensation cycle.

Subgroup analyses revealed nuanced treatment effectiveness. Conventional core stability exercises showed modest pain reduction (SMD = −0.36) and may suit long-term stability maintenance, whereas dynamic interventions like SET demonstrated superior short-term analgesic effects. This differential efficacy reflects distinct neurophysiological mechanisms: static exercises enhance *γ*-motor neuron recruitment through low-load, high-repetition protocols, while dynamic interventions promote comprehensive neuromuscular coordination.

As systematically reviewed by Lin et al. (2024) across 26 RCTs, core stability training consistently improves core muscle strength and activation, though pain relief outcomes vary, suggesting treatment responses are significantly influenced by intervention intensity and baseline patient characteristics.

For optimal clinical implementation, core training protocols should be individualized. Acute pain patients may benefit from reduced loading and shorter sessions (≤30 min), while chronic cases typically require progressive intensity escalation to induce structural adaptations. As a foundational element in CNLBP rehabilitation, core training should be strategically combined with dynamic modalities to address both immediate symptom relief and long-term functional restoration.

### Sling exercise therapy

Sling exercise therapy has emerged as an effective neuromuscular activation technique for CNLBP. Our analysis demonstrates SET's superior efficacy in pain relief (SMD = −1.43) and functional improvement (SMD = −2.60), attributable to deep muscle activation through unstable surfaces. Dynamic suspension exercises enhance spine-pelvis-hip kinetic chain efficiency by integrating trunk rotation with pelvic control, mimicking functional movements. The unstable environment promotes proprioceptive input via cerebellar-vestibular-spinal pathways, facilitating motor cortex reorganization and potentially alleviating central sensitization. Li et al. (2022) used transcranial magnetic stimulation in 30 subjects, revealing that 2-week SET normalized motor cortex topography and increased multifidus motor-evoked potential amplitude by 25%, suggesting SET disrupts the pain-motor control cycle through central neural adaptation. However, substantial heterogeneity (*I*^2^ = 86%–97%) highlights the need for protocol standardization, particularly regarding resistance loading vs. static maintenance intensities. Future studies should establish clear progression criteria to enhance clinical reproducibility.

### Pilates

Pilates has gained recognition as a mind-body exercise focusing on core control and postural alignment in CNLBP rehabilitation. While demonstrating rapid analgesic effects (SMD = −1.48), its limited functional improvement (SMD = −0.78) reflects inherent design limitations: whole-body coordination and respiratory integration reduce muscle tension, but insufficient endurance loading on local stabilizers may restrict structural recovery. The mind-body component may modulate pain perception through vagal activation and reduce kinesiophobia. Vera-Saura et al. (2024) compared standard Pilates (*n* = 33) vs. verbally-guided Pilates (*n* = 34) in 67 patients. While both groups showed comparable pain and function outcomes, the guided group demonstrated greater improvement in fear-avoidance beliefs, suggesting psychological components may enhance long-term adherence without addressing structural deficits. To address these limitations, Pilates can be augmented with resistance elements or suspension components. Luo et al. (2019) demonstrated this in a 64-patient RCT, where Pilates combined with SET outperformed SET alone after 4 weeks, showing 35% greater VAS reduction and 28% better ODI improvement, indicating enhanced short-term efficacy through combined stability and compliance benefits.

### Breathing training

Respiratory training shows promise as adjunct therapy, with moderate effects on pain (SMD = −0.75) and function (SMD = −0.87). Diaphragmatic breathing enhances intra-abdominal pressure through coordinated diaphragm-transversus abdominis contraction, particularly benefiting patients with abnormal breathing patterns. The diaphragm's multi-directional movement activates deep stabilizers, optimizing load distribution across lumbar muscles. Additionally, slow abdominal breathing may modulate visceral-somatic pain interaction via vagus-mediated gut-brain axis pathways, reducing sympathetic tone and anxiety-related muscle tension while disrupting the fear-avoidance cycle. Inspiratory muscle training may further modify pain gate control mechanisms, providing additional analgesic benefits. These multimodal mechanisms position respiratory training as a valuable complementary approach.

### Subgroup analyses: modality, combination, and duration

Subgroup analyses revealed distinct efficacy patterns among core training modalities, with SET and Pilates demonstrating the most robust pain relief effects (SMD = −1.43 and −1.48, respectively). These strong effect sizes likely stem from superior capacity for deep core muscle activation and precise neuromuscular control modulation. Conventional core stability training showed non-significant pooled effects (SMD = −0.36, *P* = 0.20) due to variability in intervention formats, while breathing training demonstrated remarkable consistency in standalone applications (SMD = −1.40, *I*^2^ = 0%), suggesting isolated respiratory training may achieve stable analgesia through optimized intra-abdominal pressure regulation—an advantage potentially diminished when combined with other therapies.

Intervention modality analysis revealed that combined approaches yielded superior functional outcomes. Core stability training paired with dynamic suspension (SMD = −1.07 vs. 0.06 for standalone) and respiratory training integrated with core exercises (SMD = −1.02) demonstrated enhanced trunk stability and functional movement control through synergistic effects. However, standalone SET showed dramatic short-term effects (SMD = −3.54), but extreme outliers contributed to excessive heterogeneity (*I*^2^ = 98%), cautioning against uncontrolled high-intensity single-modality protocols. Similarly, standalone Pilates results were compromised by extreme values, whereas combined protocols integrating aerobic or cognitive-behavioral elements demonstrated more reliable moderate effects.

Intervention duration analysis provided nuanced insights into temporal efficacy patterns. Significant pain reduction from short-term (<8 weeks) SET and Pilates (SMD range: −1.24 to −1.86) supports their use in acute phase management, while long-term (≥8 weeks) data revealed that sustained benefits from SET (SMD = −1.65) and breathing exercises (SMD = −0.94) depend more on neuroadaptive mechanisms like respiratory pattern remodeling. For functional improvement, short-term SET's extreme effects (SMD = −2.37) may reflect rapid core activation advantages, whereas long-term intervention heterogeneity from outliers exposes critical gaps in load progression protocols and outcome assessment standardization.

### Comparison with previous systematic reviews

Our findings are consistent with and extend recent meta-analyses in this field. A comprehensive meta-analysis by Guo et al. (2025) including 57 RCTs with 7,705 participants reported that core training significantly reduced pain in CNLBP patients, with Pilates showing optimal effects for pain relief (SMD = 0.75) and core resistance training for functional improvement (SMD = 0.76) (51). These results align with our findings that Pilates demonstrated strong analgesic effects (SMD = −1.48) and combined interventions yielded superior functional outcomes.

For breathing training, our pooled effect for pain (SMD = −0.75) is comparable to a meta-analysis of 13 RCTs reporting SMD = −0.84 (95% CI: −1.24 to −0.45) for pain and SMD = −0.74 (95% CI: −0.95 to −0.54) for function (52). Another recent meta-analysis similarly found that respiratory muscle training significantly reduced pain intensity (SMD = 0.77) and lumbar disability (SMD = 0.55) in individuals with low back pain (53).

Regarding sling exercise therapy (SET), previous meta-analyses have demonstrated that SET is more effective than motor control training for pain reduction (MD = −4.13) and disability (MD = −3.19) (4). Our results extend these findings by showing that SET produces particularly strong effects for functional improvement (SMD = −2.60), especially when combined with other modalities.

A network meta-analysis by Li et al. (2023) comparing 20 exercise interventions reported that Pilates (SMD = −1.52) and sling exercise (SMD = −1.19) showed superior pain improvement compared to conventional rehabilitation (54), corroborating our modality-specific findings.

### Clinical significance of findings

Beyond statistical significance, the clinical relevance of our findings warrants consideration. According to Cohen's benchmarks, standardized mean differences of 0.2, 0.5, and 0.8 represent small, moderate, and large effects, respectively. By this criterion, the overall effects for pain (SMD = −0.95) and function (SMD = −1.09) represent large clinically meaningful improvements. For specific modalities, SET and Pilates achieved very large analgesic effects (SMD > 1.4), suggesting these interventions can produce clinically noticeable pain relief that patients are likely to perceive as beneficial.

The minimal clinically important difference (MCID) for common outcome measures provides additional context. For the Oswestry Disability Index (ODI), the MCID is typically 6–10 points (or 30% improvement from baseline). For the Visual Analogue Scale (VAS), the MCID is approximately 15–20 mm on a 100 mm scale (51). While our pooled SMDs suggest substantial benefits, the heterogeneous outcome measures across studies precluded direct assessment of MCID achievement. However, the large effect sizes observed for SET and combined interventions suggest that many patients likely achieved clinically meaningful improvements.

From a clinical perspective, our findings support a stratified approach to intervention selection. For patients seeking rapid pain relief, SET or Pilates may be prioritized, particularly in short-term interventions (<8 weeks). For functional recovery, combined interventions integrating SET with other modalities appear most beneficial. The substantial heterogeneity observed across studies underscores the need for individualized prescription based on patient characteristics, preferences, and treatment goals.

### Limitations

This study has several limitations. Substantial heterogeneity (*I*^2^> 90%) persisted across analyses. Meta-regression to explore potential moderators—intervention duration, training frequency, and baseline pain severity—was precluded by inconsistent reporting of these parameters in primary studies (e.g., many trials failed to specify exact training frequency or provide baseline pain data suitable for such analysis). This underscores the need for future RCTs to adhere to standardized reporting guidelines (e.g., CERT) to enable more sophisticated analyses.

Heterogeneity in intervention parameters (frequency, intensity, adjunct therapies) highlights the necessity of standardized FITT (Frequency, Intensity, Time, Type) protocols (12). Extreme effect sizes warrant robust sensitivity analyses or Bayesian modeling approaches in future research. The synergistic mechanisms of combined interventions require elucidation through electromyography and functional imaging studies. Despite these limitations, our findings offer clinical guidance: prioritizing SET or breathing training for pain management while integrating dynamic core exercises for functional recovery, with careful adaptation to patient tolerance. Future investigations should emphasize long-term follow-up and mechanistic studies to establish evidence-based multidimensional CNLBP rehabilitation frameworks.

## Data Availability

The original contributions presented in the study are included in the article/Supplementary Material, further inquiries can be directed to the corresponding author.
